# Temporal Proteomic Analysis of Herpes Simplex Virus 1 Infection Reveals Cell-Surface Remodeling via pUL56-Mediated GOPC Degradation

**DOI:** 10.1016/j.celrep.2020.108235

**Published:** 2020-10-06

**Authors:** Timothy K. Soh, Colin T.R. Davies, Julia Muenzner, Leah M. Hunter, Henry G. Barrow, Viv Connor, Clément R. Bouton, Cameron Smith, Edward Emmott, Robin Antrobus, Stephen C. Graham, Michael P. Weekes, Colin M. Crump

**Affiliations:** 1 Department of Pathology, University of Cambridge, Cambridge CB2 1QP, UK; 2 Cambridge Institute for Medical Research, University of Cambridge, Cambridge CB2 0XY, UK

**Keywords:** herpesvirus, virus-host interaction, immune evasion, membrane trafficking, proteasomal degradation, quantitative proteomics, uncharacterized ORF, FIG, CAL, PIST

## Abstract

Herpesviruses are ubiquitous in the human population and they extensively remodel the cellular environment during infection. Multiplexed quantitative proteomic analysis over the time course of herpes simplex virus 1 (HSV-1) infection was used to characterize changes in the host-cell proteome and the kinetics of viral protein production. Several host-cell proteins are targeted for rapid degradation by HSV-1, including the cellular trafficking factor Golgi-associated PDZ and coiled-coil motif-containing protein (GOPC). We show that the poorly characterized HSV-1 pUL56 directly binds GOPC, stimulating its ubiquitination and proteasomal degradation. Plasma membrane profiling reveals that pUL56 mediates specific changes to the cell-surface proteome of infected cells, including loss of interleukin-18 (IL18) receptor and Toll-like receptor 2 (TLR2), and that cell-surface expression of TLR2 is GOPC dependent. Our study provides significant resources for future investigation of HSV-host interactions and highlights an efficient mechanism whereby a single virus protein targets a cellular trafficking factor to modify the surface of infected cells.

## Highlights

• Multiplexed proteomic screens reveal regulation of host protein abundance by HSV-1
• HSV-1 pUL56 targets host proteins such as GOPC for proteasomal degradation
• HSV-1-mediated degradation of GOPC remodels the plasma membrane of infected cells
• GOPC is important for cell-surface expression of immune receptor TLR2 in keratinocytes

## Graphical Abstract

**Figure:**
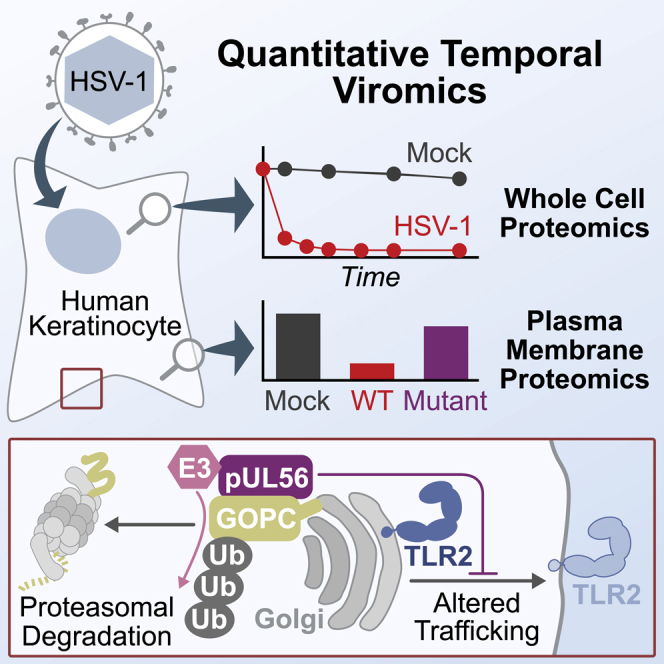

## Teaser

Soh et al. use quantitative mass spectrometry techniques to investigate how HSV-1 regulates the infected-cell proteome. They identify HSV-1 pUL56 as a ubiquitin ligase adaptor that promotes degradation of the cellular trafficking factor GOPC, lowering the abundance of immune signaling molecules on the surface of infected cells.

## Introduction

Herpesviruses are ubiquitous in the human population and are characterized by an ability to establish lifelong infections. Greater than two-thirds of the world’s population are estimated to be infected with herpes simplex virus 1 (HSV-1) or HSV-2 (Looker et al., 2008, 2015). These infections are generally asymptomatic or give rise to mild symptoms following viral reactivation (oral or genital sores), although they can cause severe diseases of the eye (herpes keratitis), central nervous system (herpes encephalitis), or systemic infections in those with compromised or immature immune systems (Gnann and Whitley, 2017; Koujah et al., 2019; Pinninti and Kimberlin, 2018).

The replication cycle of herpesviruses entails a complex and carefully controlled transcriptional cascade of viral genes that function both to generate infectious particles and to modulate host factors. HSV-1 genes are conventionally separated into three broad temporal classes (immediate early, early, and late), where proteins expressed earliest during infection serve as transcription factors and/or modulate the host-cell environment and immune responses, whereas those expressed late are structural components of the virion. The best-studied HSV-1 immunomodulatory proteins are infected-cell protein 0 (ICP0) and virion host shutoff protein (vhs). These proteins are known to change the host-cell proteome by suppressing the expression and/or promoting the degradation of various host proteins (Boutell et al., 2011; Chelbi-Alix and de Thé, 1999; Jiang et al., 2016; Lees-Miller et al., 1996; Lilley et al., 2011; Orzalli et al., 2013; Su and Zheng, 2017; Zenner et al., 2013). However, the global temporal effects of HSV-1 replication on the host proteome remain poorly characterized. To date, there has been one large-scale proteomic analysis of HSV-1 infection. This work, performed in fibroblasts, quantified the abundance of approximately 4,000 host proteins and characterized changes in protein post-translational modification following infection (Kulej et al., 2017). However, the molecular mechanisms underlying these changes were not characterized.

Quantitative temporal viromics (QTV) is a method to enable highly multiplexed quantitative analysis of temporal changes in host and viral proteins throughout the course of a productive infection (Weekes et al., 2014). QTV employs tandem mass tags (TMTs) and triple-stage mass spectrometry (MS3) to facilitate precise quantitation of each protein, and we have applied this technique to study several viruses including human cytomegalovirus (HCMV), Epstein-Barr virus, vaccinia virus, and BK polyomavirus (Caller et al., 2019; Ersing et al., 2017; Soday et al., 2019; Weekes et al., 2014).

We have now performed QTV analysis throughout a single replication cycle of HSV-1 in human keratinocytes, the natural target of HSV-1 lytic infection. At each time point, we quantified almost 7,000 human proteins and >90% of canonical HSV-1 proteins, and we have found evidence for the expression of 17 additional HSV-1 proteins beyond the canonical open reading frames (ORFs). We have identified host proteins that are rapidly degraded by HSV-1, including the cellular trafficking factor Golgi-associated PDZ and coiled-coil motif-containing protein (GOPC). Further, we demonstrate that GOPC degradation is mediated by the poorly characterized HSV-1 pUL56. Plasma membrane profiling shows that pUL56 reduces the cell-surface abundance of multiple host proteins, including the immune signaling molecule Toll-like receptor 2 (TLR2), and we demonstrate that cell-surface expression of TLR2 requires GOPC. This highlights an unanticipated and highly efficient mechanism whereby HSV-1 specifically targets a cellular trafficking factor in order to manipulate the abundance of host proteins on the surface of infected cells.

## Results

### QTV Study of HSV-1 Infection

**Figure 1:**
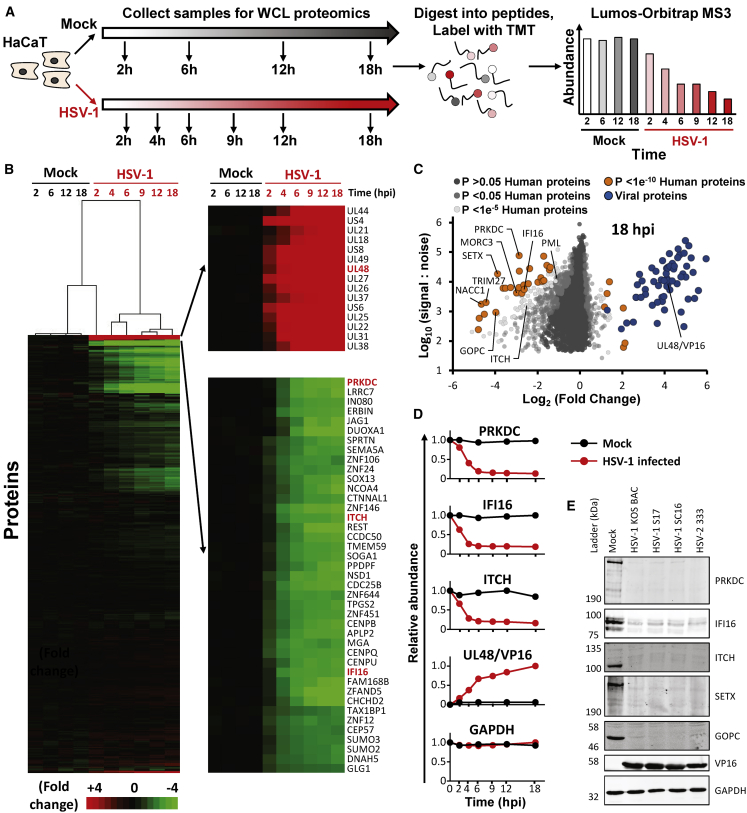
Quantitative Temporal Analysis of HSV-1 Infection (A) Schematic of the experimental workflow. HaCaT cells were infected at an MOI of 10 or mock infected. Whole cell lysate (WCL) samples were harvested at the stated times and processed for quantitative proteomic analysis. Data shown in Tables S1 and S7. (B) Hierarchical cluster analysis of all proteins quantified. An enlargement of two subclusters is shown in the right panel, including multiple proteins that were substantially up- or downregulated. (C) Scatterplot of all proteins quantified at 18 hpi. Fold changes were calculated for each protein by comparing signal:noise (S:N) values from each HSV-1-infected sample to the average S:N for that protein from the four mock-infected samples. Benjamini-Hochberg-corrected significance B was used to estimate p values. This metric calculates the probability of obtaining a log-fold change of at least a given magnitude under the null hypothesis that the distribution of log ratios has normal upper and lower tails. Two modifications are included: (1) that the spread of up- and downregulated values can be different (which can occur, for example, where multiple proteins are downregulated in the context of host shutoff); and (2) values are calculated for consecutive protein subsets obtained by sequential S:N binning, because the spread of fold-change ratios for proteins quantified by peptides with high S:N values is naturally smaller than the spread of ratios for proteins less well quantified with lower total S:N values (Cox and Mann, 2008). (D) Example temporal profiles for control proteins that are known to be degraded. (E) Validation of temporal profiles shown in (D) by immunoblot of lysates from HaCaT cells infected with HSV-1 strains KOS, S17, and SC16 and HSV-2 strain 333 (all at MOI 5) for 16 h.

To construct an unbiased global picture of changes in host and viral proteins throughout the course of HSV-1 infection, we infected an immortalized human keratinocyte cell line (HaCaT) with HSV-1 at a high multiplicity of infection (MOI; 10 plaque-forming units [PFUs]/cell) (Figure 1; Table S1). Immunofluorescence analysis of parallel samples confirmed that >95% of cells were infected (Figure S1A). Ten-plex TMTs and MS3 were used to quantify changes in protein expression over six time points (Figure 1A). A particular advantage of such TMT-based quantitation is the measurement of each protein at every time point. This generated the most complete proteomic dataset examining the lytic replication cycle of HSV-1 to date, quantifying 6,956 human proteins and 67/74 canonical HSV-1 proteins, and provided a global view of changes in protein expression during infection.

Temporal analysis of viral protein expression over the whole course of infection can provide a complementary system of protein classification, in addition to enabling direct correlation between viral and cellular protein profiles to give insights into viral-host protein interaction (Soday et al., 2019; Weekes et al., 2014). The number of classes of viral protein expression was determined by clustering viral proteins using the K-means method. This identified at least five distinct temporal profiles of viral protein expression (Figures S1B–S1E; Table S1). Furthermore, by searching data against a 6-frame translation of the HSV-1 strain used (KOS), eight putative additional HSV-1 proteins (6FT-ORFs) that increased in abundance over the course of infection were identified in this dataset (Figure S2A; Table S1).

**Figure 2:**
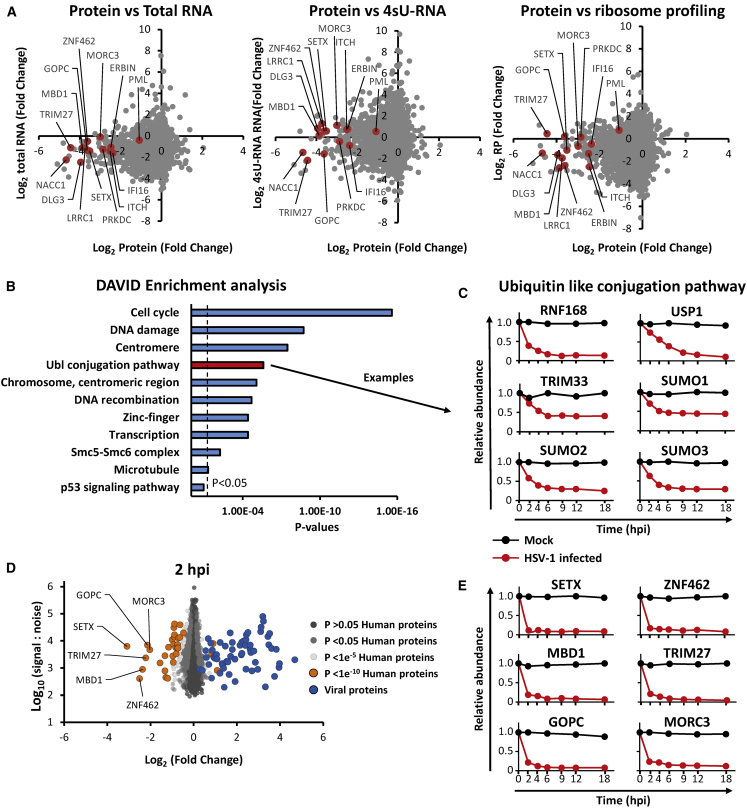
Manipulation of Host-Cell Pathways during HSV-1 Infection (A) Scatterplots comparing the fold change of protein abundance to total RNA (left), newly synthesized RNA (4sU-RNA; middle), and ribosome profiling (RP; right) data in cells infected with HSV-1 versus mock-infected cells. RNA and RP values are from Rutkowski et al. (2015). Data shown in Table S2. (B) DAVID enrichment analysis of all human proteins downregulated >2-fold at any point during infection compared to an average of the four mock samples. A background of all 6,956 quantified human proteins was used. Shown are representative terms from each cluster with Benjamini-Hochberg-corrected p values of <0.05. Components of each enriched cluster are shown in Table S3. A similar analysis was performed for proteins upregulated >2 fold; however, this did not reveal any significant enrichment. (C) Example temporal profiles of proteins downregulated from the ubiquitin-like (Ubl) conjugation pathway. (D) Scatterplot of all proteins quantified at 2 hpi. Fold changes were calculated for each protein by comparing the S:N value from the 2-hpi HSV-1-infected sample to the average S:N for that protein from the four mock-infected samples. Benjamini-Hochberg-corrected significance B was used to estimate p values (Cox and Mann, 2008). (E) Temporal profiles of all proteins downregulated during HSV infection >4-fold at 2 hpi.

HSV-1 infection led to >2-fold downregulation of 496 human proteins and >2-fold upregulation of 34 human proteins. Mock and immediate-early (2 h) infection samples clustered separately from early (4, 6 h) and late (9, 12, 18 h) infection time points. The most extensive changes to the cellular proteome occurred late during infection, as might be expected for a virus with a potent host shutoff activity (Figure 1B). This effect can be observed by a general shift to the left in a scatterplot of fold change (Figure 1C). Multiple host targets known to be specifically downregulated during HSV-1 infection were confirmed, including DNA-PKcs (PRKDC) (Lees-Miller et al., 1996; Parkinson et al., 1999), interferon gamma-inducible protein 16 (IFI16) (Orzalli et al., 2012), itchy E3 ubiquitin protein ligase (ITCH) (Ushijima et al., 2010), promyelocytic leukemia (PML) (Chelbi-Alix and de Thé, 1999), tripartite motif-containing 27 (TRIM27) (Conwell et al., 2015), nucleus accumbens-associated 1 (NACC1) (Sloan et al., 2015), and MORC family CW-type zinc-finger 3 (MORC3) (Sloan et al., 2015) (Figures 1C, 1D, and 2D; Table S1). Proteomic data were validated by comparison to immunoblot analysis of cells infected for 16 h with three independent strains of HSV-1 and with HSV-2, which suggested that many of the changes observed were conserved phenotypes (Figure 1E). All data are shown in Table S1, in which the “plotter” worksheet facilitates interactive generation of temporal graphs of expression of each of the human or viral proteins quantified. Our data on HSV-1-dependent changes to the cellular proteome were compared to data on HSV-1-dependent changes to the transcriptome (total and newly synthesized RNA) and translatome (ribosome profiling) from a recent study (Rutkowski et al., 2015) using the latest time points from each dataset to compare the greatest abundance changes (18 h for the proteome and 8 h for the transcriptome/translatome; Table S2). These data confirm a general decrease in both protein and total RNA abundance (Figure 2A). However, the data also suggest the proteins exhibiting the largest decreases in abundance are targeted for specific HSV-1-induced protein degradation, rather than inhibition of transcription or translation (Figure 2A). For example, in HSV-1-infected cells the protein TRIM27 was 22-fold less abundant but TRIM27 total RNA was only 2.2-fold reduced, newly synthesized RNA was just 4.4-fold reduced, and there was a slight increase in ribosome-protected fragments. Table S2 shows the comparison of protein abundance changes at 18 h post infection (hpi) versus total RNA, newly synthesized RNA (4sU), and ribosome profiling data (from Rutkowski et al., 2015), including a plotter function for host proteins quantified across all four datasets.

### Bioinformatic Enrichment Analysis of HSV-1 Infection

DAVID software (Huang et al., 2009) was used to identify pathways significantly enriched among proteins downregulated >2-fold (Figure 2B). Several of these pathways are known to influence HSV-1 infection, for example cell-cycle-associated proteins such as cyclin-dependent kinases (Schang et al., 1998) and a range of DNA damage response pathways (reviewed in Smith and Weller, 2015). The ubiquitin-like (Ubl) conjugation pathway was significantly enriched, consistent with the known targeting of certain pathway components by herpesviruses to direct cellular prey for degradation. For example, three SUMO family members were downregulated during infection (the fourth was not quantified) (Figure 2C). Components of each enriched cluster are shown in Table S3. A similar analysis of host proteins upregulated >2-fold did not reveal any enriched clusters.

### Identification of Host Targets Most Rapidly Depleted following HSV-1 Infection

Based on the premise that host proteins downregulated early during viral infection are likely to be enriched in factors with antiviral activity (Nightingale et al., 2018), we analyzed proteins downregulated >4-fold at the earliest time point after HSV-1 infection (2 hpi; Figures 2D and 2E). Of the six proteins thus identified, four have previously been shown to be reduced significantly in HSV-1-infected cells (methyl-CpG-binding domain protein 1 [MBD1] [Sloan et al., 2015], MORC3 [Sloan et al., 2015], TRIM27 [Conwell et al., 2015], and zinc-finger protein 462 [ZNF462] [Sloan et al., 2015]), of which three were shown to be modulated in an ICP0-dependent manner (MBD1, MORC3, and TRIM27) (Sloan et al., 2015). The other two proteins (senataxin [SETX] and GOPC) have not been previously identified as targets of HSV-1-mediated degradation.

### pUL56 Binds the NEDD4 Family of Ubiquitin Ligases and GOPC

**Figure 3:**
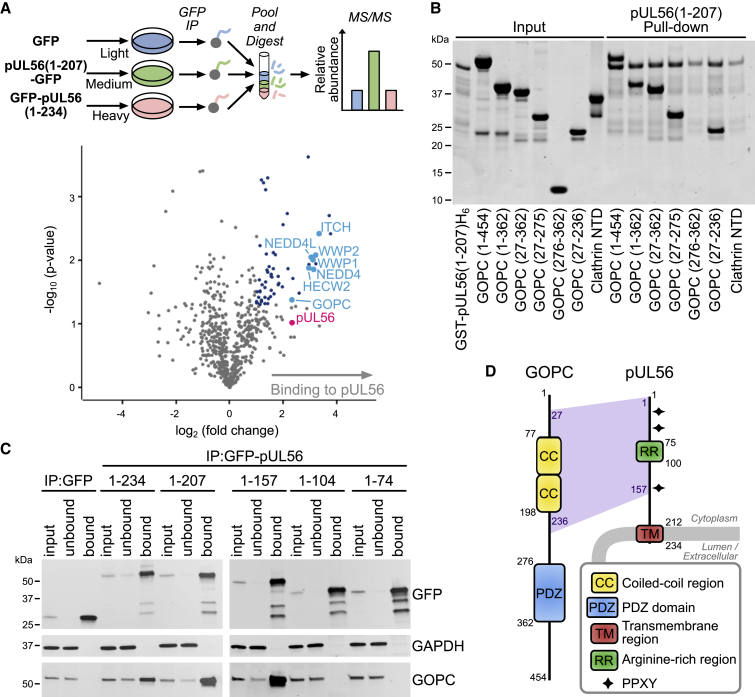
pUL56 Binds GOPC and Cellular Ubiquitin Ligases (A) SILAC-labeled HEK293T cells were transfected with GFP-tagged pUL56 cytoplasmic domain (residues 1–207) or GFP alone and subjected to immunoprecipitation (IP) using a GFP affinity resin. In the volcano plot, the horizontal axis shows average fold enrichment in IP of pUL56(1–207)-GFP compared to GFP across three biological replicates and the vertical axis shows significance (two-sided t test) across the three replicates. Significantly enriched proteins (>2-fold enrichment and p < 0.05) are colored blue and selected proteins are annotated. Data shown in Table S4. (B) Pull-down experiment using purified recombinant components, demonstrating that the GST-tagged pUL56 cytoplasmic domain interacts directly with the coiled-coil region of GOPC. The peptide-binding N-terminal domain of clathrin heavy chain (Clathrin NTD) and GST were used as control prey and bait proteins, respectively. Proteins were visualized using InstantBlue Coomassie stain. (C) CoIP of GOPC with GFP-tagged pUL56 and truncations thereof. Immunoblots were stained with the antibodies shown. (D) Schematic representation of pUL56 and GOPC.

ITCH, a member of the NEDD4 family of ubiquitin ligases, was rapidly depleted during HSV-1 infection (Figures 1B–1E). pUL56 proteins from HSV-1 and HSV-2 interact with ITCH and NEDD4, leading to proteasomal degradation of these targets (Ushijima et al., 2008, 2010). pUL56 is a tail-anchored type II membrane protein found in purified virions (Koshizuka et al., 2002) and contains three PPXY motifs that interact with NEDD4, likely by binding to WW domains (Ushijima et al., 2008). Notably, pUL56 does not contain any lysine residues and is thus likely to be refractory to ubiquitination. To further characterize the cellular binding partners of pUL56, stable isotope labeling of amino acids in cell culture (SILAC) immunoprecipitation-mass spectrometry (IP-MS) analysis was performed using cells expressing GFP-tagged pUL56 or GFP alone (Figures 3A and S3; Table S4). Several members of the NEDD4 family of ubiquitin ligases were enriched in the pUL56 IP, as were multiple trafficking protein particle complex II (TRAPPCII) subunits. Strikingly, GOPC was also identified as a binding partner of pUL56. Co-precipitation assays demonstrated that the purified glutathione S-transferase (GST)-tagged pUL56 cytoplasmic domain (residues 1–207) is capable of binding purified GOPC, confirming that these two proteins interact directly (Figure 3B). The N-terminal coiled-coil domain of GOPC mediates its recruitment to the Golgi via an interaction with golgin-160 (Hicks and Machamer, 2005), whereas the PDZ domain mediates interactions with C-terminal PDZ-binding motifs of cellular partner proteins (Yao et al., 2001). Truncation of GOPC showed that residues 27–236, comprising the N-terminal coiled-coil region, are sufficient to bind to pUL56 (Figure 3B). IP experiments conducted with cells expressing truncated forms of pUL56 demonstrated that residues 1–157 of pUL56 can mediate efficient binding to GOPC whereas residues 1–104 do not, suggesting that a binding site for GOPC may reside within the 53-amino acid sequence between pUL56 residues 105 and 157 (Figure 3C). Taken together, these results suggest a model whereby pUL56 binds both GOPC and the NEDD4 family of ubiquitin ligases, bringing them in close proximity and thus stimulating the ubiquitination and proteolytic degradation of GOPC.

### pUL56 Mediates Degradation of GOPC via the Proteasome

**Figure 4:**
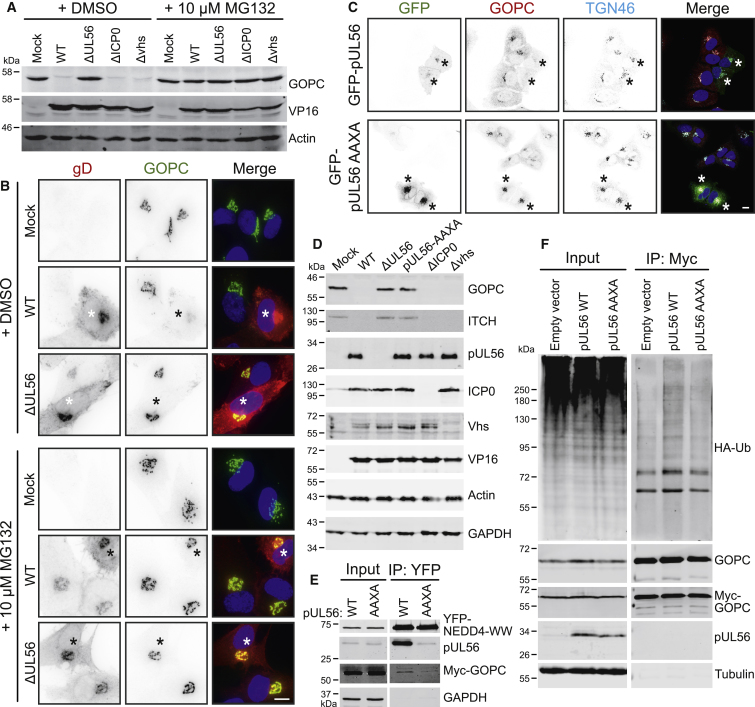
pUL56 Is Necessary and Sufficient for GOPC Degradation (A) HaCaT cells were infected at an MOI of 10 with the indicated viruses. After 2 h, media were replaced with 10 μM MG132 or carrier (DMSO) in DMEM for the remainder of the infection. Cell lysates were harvested 16 hpi and the indicated proteins were detected by immunoblot. (B) HFF hTERT cells were infected at an MOI of 1 and then treated with MG132 or carrier as described in (A). At 6 hpi, samples were fixed and stained for GOPC (green) and the infection control gD (red). The merge includes DAPI (blue). The scale bar represents 10 μm. Asterisks indicate HSV-1 infected (gD expressing) cells. (C) U2-OS cells were transfected with GFP-pUL56 or GFP-pUL56-AAXA expression plasmids. One day post-transfection, cells were fixed and stained for GOPC (red) and TGN46 (cyan). The merge includes DAPI (blue). The scale bar represents 10 μm. Asterisks indicate GFP-pUL56 and GFP-pUL56 AAXA expressing cells. (D) HaCaT cells were infected at an MOI of 10 with the indicated virus, cell lysates were harvested 16 hpi, and the indicated proteins were detected by immunoblot. (E) HEK293T cells were transfected with YFP-tagged NEDD4-WW domains, myc-tagged GOPC, and untagged pUL56 or pUL56-AAXA expression plasmids. Samples were subjected to IP using YFP affinity resin and co-precipitated proteins were detected by immunoblot. (F) HEK293T cells were transfected with HA-tagged ubiquitin (HA-Ub) and myc-GOPC together with empty vector or pUL56 or pUL56-AAXA expression plasmids. Samples were subjected to IP using myc affinity resin and probed for the presence of HA-Ub-conjugated GOPC by immunoblot.

To identify the mechanism of GOPC degradation, cells were infected with wild-type (WT) HSV-1 or HSV-1 lacking expression of pUL56 (ΔUL56). Viruses lacking expression of the viral proteins ICP0 (ΔICP0) or vhs (Δvhs) were also included, as both are known to deplete host proteins. Cells were further treated with or without the proteasomal inhibitor MG132. GOPC was degraded during HSV-1 infection in a pUL56-dependent and MG132-inhibitable fashion, whereas GOPC degradation was independent of both ICP0 and vhs (Figure 4A). Immunofluorescence microscopy further demonstrated pUL56-dependent loss of GOPC in HSV-1-infected cells, which was inhibited by MG132 (Figure 4B). HSV-1 pUL56 contains three PPXY motifs, which mediate interaction with the NEDD4 family of E3 ubiquitin ligases (Ushijima et al., 2010). Expression of a GFP-tagged construct by transfection demonstrated that pUL56 is sufficient to cause GOPC degradation in the absence of other HSV-1 factors (Figure 4C). Furthermore, the degradation of GOPC was shown to rely on the PPXY motifs of pUL56, as GOPC was not depleted in cells expressing GFP-tagged pUL56 where all three PPXY motifs have been mutated to AAXA (GFP-pUL56-AAXA; Figure 4C). GFP-pUL56-AAXA simultaneously co-localized with GOPC and TGN46 at a juxtanuclear compartment, suggesting pUL56 and GOPC interact at Golgi membranes, where both proteins are known to localize (Hicks and Machamer, 2005; Koshizuka et al., 2002). To further test the importance of NEDD4 family E3 ubiquitin ligase binding for GOPC degradation by pUL56, a recombinant virus was generated where all three pUL56 PPXY motifs were mutated to AAXA. This mutant phenocopied the pUL56-deletion virus, failing to degrade GOPC and ITCH (a known pUL56 target; Ushijima et al., 2010), even though pUL56 expression was maintained (Figure 4D). To test our model of pUL56 binding simultaneously to GOPC and NEDD4 family E3 ubiquitin ligases, untagged pUL56 (WT or AAXA) was co-expressed with myc-tagged GOPC plus yellow fluorescent protein (YFP)-tagged WW domains of NEDD4, which interact with PPXY motifs, and cell lysates were subjected to IP analysis with a YFP affinity resin. Capture of the YFP-NEDD4-WW domains efficiently co-precipitated WT pUL56 but not pUL56-AAXA (Figure 4E). Importantly, myc-GOPC was co-precipitated with YFP-NEDD4-WW in the presence of WT pUL56, demonstrating formation of a tripartite complex where binding of GOPC to NEDD4 is mediated by pUL56. Furthermore, IP experiments conducted with cells expressing myc-GOPC and hemagglutinin (HA)-tagged ubiquitin demonstrated a marked increase in ubiquitinated myc-GOPC species precipitated from cells co-expressing WT pUL56 as compared to pUL56-AAXA (Figure 4F). Overall, these data demonstrate that pUL56 recruits NEDD4 family ubiquitin ligases to mediate the ubiquitination and proteasomal degradation of GOPC.

### Replication of HSV-1 in Cell Culture Is Independent of pUL56

**Figure 5:**
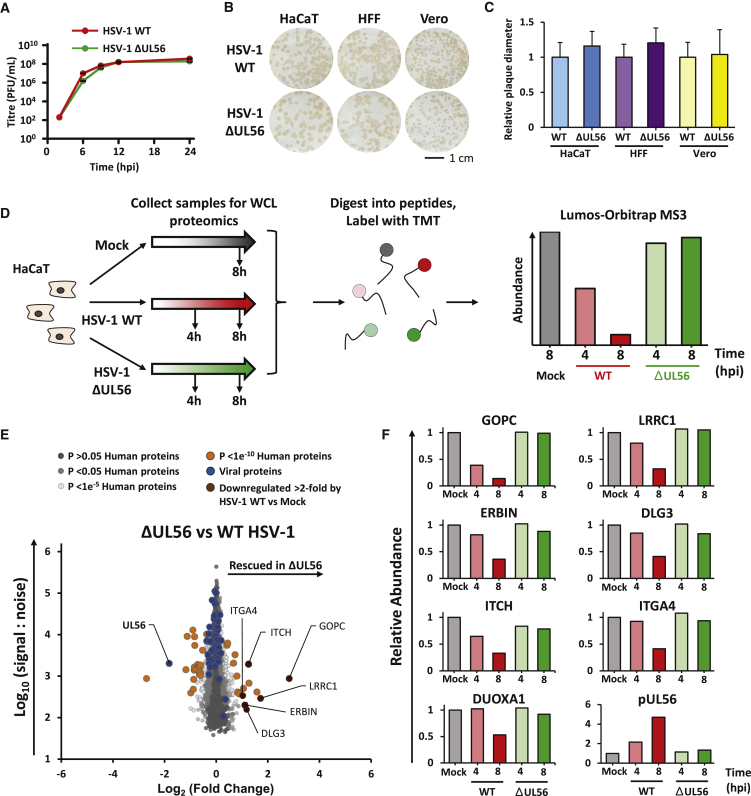
Identification of pUL56 Degradation Targets (A) HaCaT cells were infected with HSV-1 WT and HSV-1 ΔUL56 at an MOI of 10 in biological duplicates and total infectious virus yields at the indicated time points were determined by plaque assay. Error bars represent standard error of the mean. (B) Plaque assays of HSV-1 WT and HSV-1 ΔUL56 in HaCaT, HFF hTERT, and Vero cells in biological duplicates. Plaques were visualized by immunostaining the cells for the viral glycoprotein gD. (C) Plaque diameters from (B) were measured and normalized to the average for HSV-1 WT. Error bars represent standard deviation; n = 35–67. (D) Schematic of the proteomics workflow. Cells were infected at an MOI of 10 or mock infected. Samples were harvested at the stated times and processed for quantitative proteomic analysis. Data shown in Table S5. (E) Scatterplot of all proteins quantified. Fold changes were calculated for each protein by comparing S:N values at 8 hpi for HSV-1-WT- and HSV-1-ΔUL56-infected samples. Benjamini-Hochberg-corrected significance B was used to estimate p values (Cox and Mann, 2008). (F) Temporal profiles of all proteins downregulated >2-fold by HSV-1 WT versus mock and additionally rescued >2-fold by HSV-1 ΔUL56.

The rapid depletion of GOPC from cells during HSV-1 infection implies that removal of this host protein may be important for efficient viral replication. However, growth kinetics of HSV-1 ΔUL56, where endogenous levels of GOPC are maintained during infection, were essentially identical to kinetics of HSV-1 WT (Figure 5A). Plaque size analysis also demonstrated no defects in cell-to-cell spread for HSV-1 ΔUL56 compared to WT (Figures 5B and 5C). These data demonstrate that pUL56 is dispensable for HSV replication in cell culture, consistent with previous reports (Ushijima et al., 2008). Given that viruses do not usually retain genes of no benefit, this suggests that pUL56 plays a role during viral replication *in vivo*, perhaps during establishment, maintenance, or reactivation from latency. Alternatively, pUL56 may be a virulence factor involved in modulating antiviral immune responses against HSV-1, as is the case for a number of herpesvirus proteins that are dispensable in cell culture but important for replication *in vivo*, for example vhs (Strelow and Leib, 1995).

### Identification of Host Proteins Specifically Depleted by pUL56

ICP0 and vhs are known to cause extensive remodeling of host protein expression to facilitate viral replication (Boutell and Everett, 2013; Smiley, 2004). Our data now suggest that pUL56 also contributes to host protein depletion but in a more targeted manner. To identify cellular proteins depleted by pUL56, HaCaT cells were infected with HSV-1 WT or ΔUL56 and analyzed by TMT-based proteomics (Figure 5D; Table S5). Of the 7,696 human proteins quantified, only a small number exhibited significant abundance changes between the WT and ΔUL56 infections, and the largest change observed was for GOPC (Figures 5E and 5F). A small number of other potential targets of pUL56 were identified, defined by >2-fold reduced abundance in HSV-1 WT samples compared to mock and ΔUL56 samples. These included discs-large MAGUK scaffold protein 3 (DLG3), leucine-rich repeat-containing 1 (LRRC1), and Erbb2-interacting protein (ERBIN), which may function as a complex: both LRRC1 (also known as LANO) and ERBIN have been shown to interact with DLG proteins (Saito et al., 2001). The DLG family has a number of proposed functions including regulation of cell polarity and tight junction formation, and they are targeted for degradation by a number of viral families (Kong et al., 2014; Lee et al., 1997; Roberts et al., 2012). Remodeling cell polarity through pUL56-mediated degradation of these host proteins may facilitate HSV-1 spread *in vivo*.

Searching this TMT dataset against a 6-frame translation of KOS-strain HSV-1 identified 14 putative additional HSV-1 proteins that increased in abundance over the course of infection, including 9 that were not identified in the initial QTV experiment (Figure S2B; Table S5). Comparison of our two MS datasets on protein abundance in HSV-1-infected cells by linear regression analysis showed close correlation (r^2^ = 0.75) between the changes caused by WT HSV-1 at 9 hpi (Table S1, dataset) and 8 hpi (Table S5, dataset), demonstrating the reproducibility of our data (Figure S4).

### pUL56 Activity Alters the Plasma Membrane Proteome

**Figure 6:**
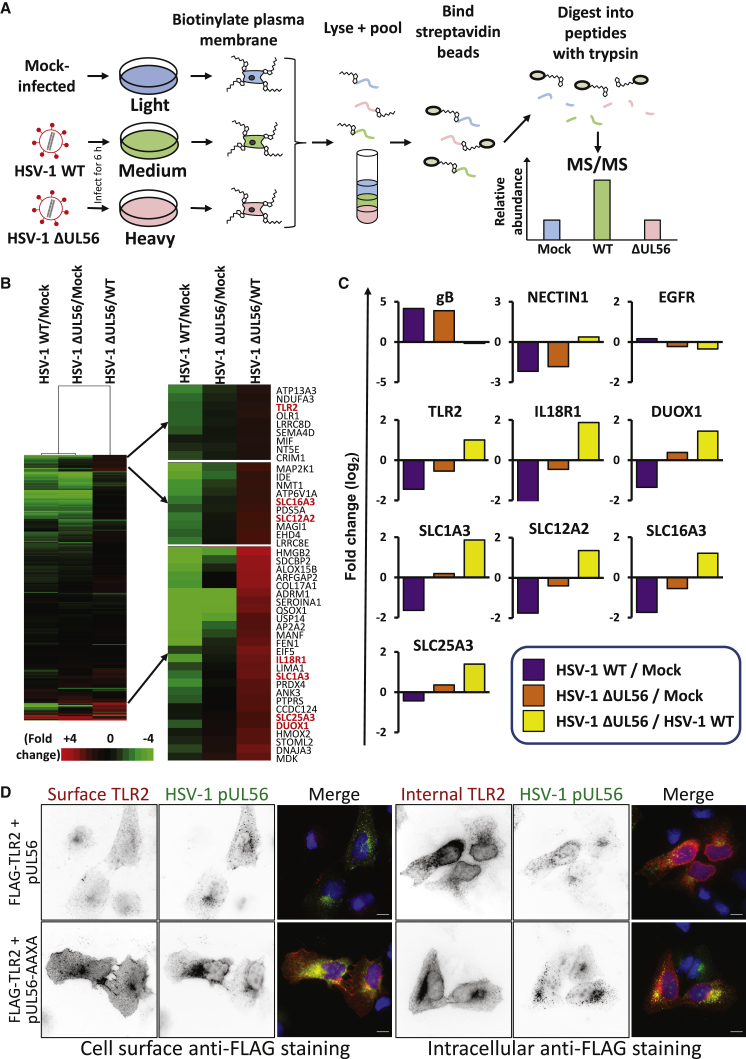
pUL56 Modulates Immune Receptors through Control of Host Trafficking to the Plasma Membrane (A) Schematic of the experimental workflow. SILAC-labeled cells were infected at an MOI of 10 or mock infected. Samples were harvested 6 hpi and processed for plasma membrane enrichment and subsequent quantitative MS. Data shown in Tables S6 and S7. (B) Hierarchical cluster analysis of fold-change values for each pairwise comparison. Proteins were included if they were annotated as plasma membrane (PM), cell surface (CS), or extracellular (XC) by Gene Ontology (GO), or with a short GO (ShG) term as previously described (Weekes et al., 2014). An enlargement of three clusters is shown in the right panel, which included proteins downregulated during infection with HSV-1 WT but rescued by infection with HSV-1 ΔUL56. (C) Profiles of example proteins that were downregulated >2-fold by HSV-1 WT and rescued >2-fold by HSV-1 ΔUL56 are shown, as well as the controls gB (expressed only in infected cells), NECTIN-1 (removed from the cell surface by HSV-1), and EGFR (unchanged). (D) U2-OS cells were transfected with FLAG-TLR2 together with pUL56 or pUL56-AAXA expression plasmids. One day post-transfection, cells were stained for surface TLR2 before fixation or intracellular TLR2 after fixation and permeabilization (both red) and co-stained for pUL56 (green). The merge includes DAPI (blue). The scale bars represent 10 μm.

Modulation of proteins at the cell surface is an immune evasion strategy utilized by multiple viruses. Because GOPC regulates the trafficking of certain proteins to the plasma membrane (Cheng et al., 2002), destruction of GOPC through the activity of pUL56 may be a mechanism to specifically modify the surface presentation of proteins in HSV-1-infected cells. Plasma membrane profiling was thus performed on cells infected with HSV-1 WT or ΔUL56 at an early stage of replication (6 hpi) using SILAC-based MS (Figure 6). Filtering for proteins annotated as plasma membrane (PM), cell surface (CS), or extracellular (XC) by Gene Ontology (GO) or with a short GO (ShG) term as previously described (Weekes et al., 2014) resulted in >700 quantified host proteins (Table S6). Hierarchical clustering of the resulting data identified host proteins that are less abundant at the plasma membrane of HSV-1-WT-infected cells and rescued by pUL56 deletion (Figure 6B). These included immune signaling proteins TLR2 and interleukin-18R1 (IL18R1) as well as DUOX1 (dual oxidase 1) and several members of the solute carrier (SLC) family of proteins (Figures 6B and 6C).

Comparison of plasma membrane and whole-cell proteomic datasets for the same time point of HSV-1 infection (6 hpi) identified 360 proteins conforming to our plasma membrane filter that were quantified in both experiments (Table S7). Interestingly, of 121 annotated plasma membrane proteins that were downregulated >2-fold by 6 h of HSV-1 infection, only 9 were also downregulated >2-fold at the same time point from whole-cell lysates (Figure S5; Table S7), including the HSV-1 receptor nectin-1 that is known to be downregulated in infected cells (Stiles et al., 2008). Given the majority of plasma membrane proteins, including major histocompatibility complex class I (MHC-I) (indicated by β2-microglobulin) and DUOX1, were downregulated substantially more from the plasma membrane than in whole-cell lysates (Figure S5; Table S7), this suggests that during HSV-1 infection these and many other plasma membrane proteins may be downregulated by intracellular sequestration as opposed to degradation or transcriptional downregulation, as previously shown for MHC-I (York et al., 1994). However, TLR2 and IL18R were only quantified in plasma membrane but not whole-cell protein samples, precluding a comparison of total abundance and cell-surface abundance for these proteins (Table S7). Therefore, we employed immunofluorescence microscopy to investigate the pUL56-dependent changes to TLR2 plasma membrane localization. These data demonstrated that expression of WT pUL56, but not pUL56-AAXA, reduced cell-surface TLR2 without affecting intracellular expression levels (Figure 6D), indicating that pUL56 modulates TLR2 subcellular localization rather than targeting it for degradation.

TLR2 is a pattern recognition receptor that has a well-established activity against bacterial pathogen-associated molecular patterns (PAMPs) but also recognizes HSV-1 and HCMV glycoproteins (Boehme et al., 2006; Cai et al., 2013; Leoni et al., 2012). In response to herpesvirus infection, TLR2 plays a role in inducing interferon γ in neurons and cytokines in peritoneal macrophages, as well as controlling viral load in the CNS (Kurt-Jones et al., 2004; Lima et al., 2010; Sørensen et al., 2008). IL18 is a proinflammatory cytokine that binds IL18R1, which is important for innate immune responses to HSV-2 infection *in vivo* (Harandi et al., 2001). Downregulating these immune receptors from the cell surface may be a proviral strategy to decrease inflammation and immune activation. DUOX1 is a transmembrane protein that can generate H_2_O_2_ and functions in lactoperoxidase-mediated antimicrobial defense at mucosal surfaces (Sarr et al., 2018). Production of H_2_O_2_ has been shown to inhibit the splicing of influenza A virus (IAV) transcripts and decrease production of infectious virus, and IAV has been shown to downregulate DUOX1 (Strengert et al., 2014). Removing DUOX1 from the plasma membrane may be similarly proviral for HSV-1 by inhibiting H_2_O_2_ production. The mechanism by which HSV-1 depletes DUOX1 from the plasma membrane may be through pUL56-dependent degradation of DUOXA1 (Figure 5F) as DUOXA1 is a chaperone required for the maturation and transport of DUOX1 from the endoplasmic reticulum (ER) to the plasma membrane (Grasberger and Refetoff, 2006).

### TLR2 Cell-Surface Expression Is GOPC Dependent

**Figure 7:**
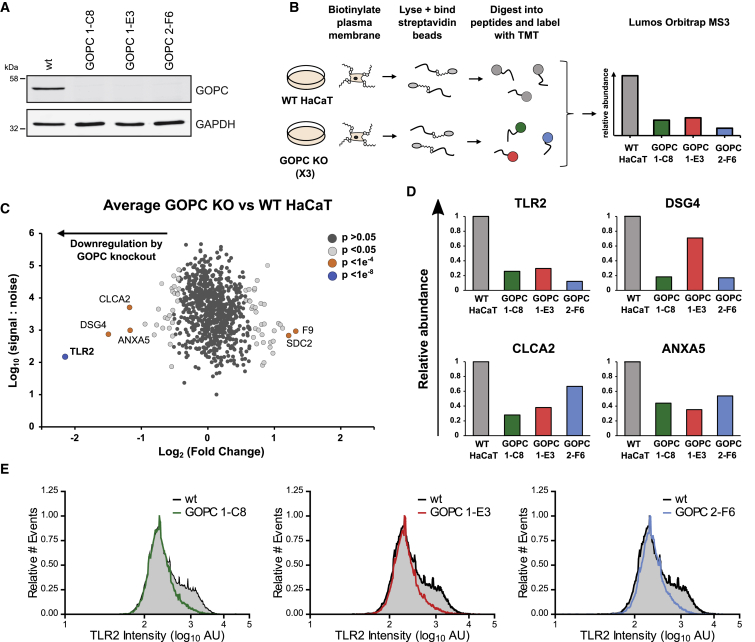
GOPC Is Important for Presentation of TLR2 at the Plasma Membrane (A) Immunoblot analysis of GOPC-knockout cells. Single-cell clones (C8, E3, and F6) were isolated from CRISPR-knockout cells made from two independent gRNAs (GOPC 1 and GOPC 2). (B) Schematic of the experimental workflow. Samples were harvested and processed for plasma membrane enrichment and subsequent TMT-based quantitative MS. Data shown in Table S8. (C) Scatterplot of all proteins annotated as PM, CS, or XC or with an shG term, comparing the average of the 3 GOPC-knockout cell lines and WT HaCaT cells. (D) Profiles of proteins that were downregulated >2-fold in the averaged GOPC-knockout cell data are shown for each independent cell line. Benjamini-Hochberg-corrected significance B was used to estimate p values (Cox and Mann, 2008). (E) Flow cytometry analysis of TLR2 levels at the plasma membrane of HaCaT WT cells and three GOPC-knockout clones (C8, E3, and F6).

To determine whether loss of TLR2 from the cell surface was due to disruption of GOPC-mediated trafficking, we generated GOPC-knockout HaCaT cells using CRISPR/Cas9 genome editing. Plasma membrane profiling was conducted on three independent single-cell knockout clones, generated from two independent single guide RNAs (sgRNAs) targeting GOPC, using TMT-based MS (Figures 7A and 7B; Table S8). A particular benefit of this approach is that although many cell-surface proteins exhibited variation in expression between the cell clones, a short list of proteins that were commonly modulated due to loss of GOPC was highlighted. Four proteins were on average downregulated >2-fold across the three independent GOPC-knockout clones, with TLR2 most substantially and consistently downregulated (Figures 7C and 7D). Scatterplots comparing individual GOPC-knockout clones to WT HaCaT cells are shown in Figure S6. Loss of TLR2 from cells lacking GOPC was further confirmed by flow cytometry. Normal HaCaT cells included a TLR2^+^ population, whereas all three GOPC-knockout clones exhibited reduced cell-surface TLR2 (Figure 7E). Taken together, these data demonstrate the expression of TLR2 on the cell surface of keratinocytes relies on the activity of GOPC.

## Discussion

In this study, we combined three powerful unbiased proteomic techniques, QTV (Figures 1, 2, and 5), affinity enrichment (Figure 3), and plasma membrane proteomics (Figures 6 and 7), to identify that HSV-1 pUL56 promotes degradation of the host-cell trafficking factor GOPC and in doing so lowers the abundance of important immune signaling molecules such as TLR2 at the plasma membrane of infected cells. Biochemistry and cell biology experiments (Figures 3, 4, 5, and 7) confirmed that pUL56 binds directly to GOPC, is both necessary and sufficient to promote GOPC degradation, requires the recruitment of the NEDD4 family of ubiquitin ligases via its PPXY motifs to stimulate GOPC ubiquitination and degradation, and results in changes in the cell-surface proteome through the loss of GOPC. The proteomic datasets presented in this manuscript represent a rich resource for identifying and characterizing the mechanisms by which HSV modulates both the whole-cell and plasma membrane proteomes of infected cells.

### Temporal Insights into HSV-1 Infection

The multiplexed quantitative proteomic data presented herein represent the most comprehensive analysis of host-cell proteome changes upon HSV-1 infection to date, with >7,000 host proteins from whole-cell samples and >700 plasma membrane proteins quantified within three independent datasets (Tables S1, S5, and S6). Comparison of our whole-cell proteomic data with published transcriptomic data and with results of our plasma membrane proteomic experiments highlighted the interesting observation that HSV-1-mediated protein downregulation from whole-cell samples appeared predominantly degradative whereas downregulation of proteins from the plasma membrane appeared primarily due to intracellular sequestration, at least for the host proteins showing the greatest depletion. These observations will need further confirmation in future studies, for example through use of protein degradation inhibitors or immunofluorescence microscopy analysis of protein localization changes in response to HSV-1 infection.

Our QTV data also provide important insights into the kinetics of HSV-1 protein production. K-means analysis identified five distinct profiles of protein expression (Figure S1). Immediate-early and early genes were found in the same class (Tp2). This presumably arises from the high MOI, required for complete infection, and the use of 2 hpi as the earliest time point. These conditions may have masked some of the differences in the kinetic profiles of immediate-early and early gene classes. Interestingly, late genes appeared to cluster in three distinct groups (temporal profile 3 [Tp3]–Tp5). Whereas late genes have previously been divided into late and true late classes, dependent on the requirement for prior genome replication (Kibler et al., 1991), our data suggest that an intermediate kinetic class may exist. Alternatively, these data may highlight differences in the translation or maturation rates of viral proteins despite their mRNA expression being induced at the same time.

This kinetic analysis of HSV-1 protein abundance also identified that ICP47 (US12) has a separate temporal profile (Tp1; Figure S1). Unlike all other viral proteins, where the abundance increases throughout infection, the amount of ICP47 peaks early during infection and the protein is subsequently downregulated. ICP47 binds and inhibits the MHC-I peptide loading complex transporter of antigenic peptides (TAP), preventing peptide presentation at the cell surface and promoting immune evasion (Hill et al., 1995). The varying abundance of ICP47 during infection might therefore have the effect of balancing evasion of CD8^+^ T cells with preventing activation of natural killer (NK) cell killing, by precisely regulating the level of MHC-I reduction at the cell surface.

### HSV-1 pUL56 Degrades GOPC by Recruiting Cellular E3 Ligases

HSV-1 strains lacking pUL56 are attenuated in animal models (Berkowitz et al., 1994; Kulej et al., 2017; Rösen-Wolff et al., 1991), despite the protein being dispensable for virus replication in cultured cells (Figures 5A–5C) (Ushijima et al., 2008). Our data provide a molecular mechanism by which pUL56 may enhance virulence during infection, by promoting the degradation of GOPC and subsequent downregulation of immune signaling molecules from the surface of infected cells.

Previous studies from HSV-1 and HSV-2 have shown pUL56 to interact with ITCH and NEDD4, leading to their degradation, but the importance of this activity remained elusive (Ushijima et al., 2008, 2010). Our IP-MS data revealed that pUL56 binds multiple cellular NEDD4 family ubiquitin ligases and the trafficking factor GOPC (Figure 3A). We show that pUL56 binds directly to the coiled-coil region of GOPC (Figure 3B), is necessary for the proteasome-mediated degradation of GOPC in HSV-1-infected cells (Figures 4A and 4B), and is sufficient to promote GOPC degradation in the absence of infection (Figure 4C). Furthermore, we show that the NEDD4-binding PPXY motifs of pUL56 are required for GOPC degradation (Figures 4C and 4D), pUL56 can simultaneously bind GOPC and NEDD4 (Figure 4E), and pUL56 can stimulate ubiquitination of GOPC (Figure 4F). Taken together, these data demonstrate that pUL56 serves as a scaffold to bring GOPC and a NEDD4 family ubiquitin ligase together in order to promote GOPC ubiquitination and proteasomal degradation. pUL56 is itself protected from degradation as it does not contain lysine residues to which ubiquitin could be conjugated, suggesting each molecule of pUL56 could turn over multiple copies of GOPC and other targets.

GOPC is rapidly degraded during HSV-1 WT infection (Figure 2D) and its abundance is restored during infection with HSV-1 ΔUL56 (Figures 4A, 4B, 5E, and 5F). Several other proteins are also rescued when comparing HSV-1 WT to ΔUL56 infection. This may reflect direct pUL56-mediated degradation or be an indirect consequence caused by the loss of GOPC.

### HSV-1 Degrades a Trafficking Factor to Modify the Surface of Infected Cells

Many viruses modify the surface of infected cells in order to modulate host responses. For example, HIV-1 Vpu recruits an E3 ubiquitin ligase to promote the ubiquitination and degradation of several cell-surface proteins (Matheson et al., 2015). Alternatively, it has been shown that multiple HCMV proteins act via distinct mechanisms to restrict the cell-surface presentation of MHC-I and NK cell receptors (Wilkinson et al., 2008). Using global unbiased approaches, we have now identified that HSV-1 pUL56 modifies the surface abundance of several host proteins including immune signaling proteins TLR2 and IL18 receptor, at least in part through specifically degrading the cellular trafficking factor GOPC. Furthermore, we have shown that the cell-surface expression of TLR2 in uninfected human keratinocytes is dependent on GOPC. This expands the known repertoire of cellular proteins whose transport is regulated by GOPC, the best characterized of which being the cystic fibrosis transmembrane regulator (Cheng et al., 2002) and G-protein-coupled receptors such as the β1-adrenergic receptor (Koliwer et al., 2015).

The roles of TLR2 during natural HSV infection are unclear, with evidence suggesting that TLR2 is important for controlling infection (Bochud et al., 2007; Sørensen et al., 2008) but also that TLR2 activation increases immunopathology in mouse models of HSV infection (Kurt-Jones et al., 2004) and pseudorabies virus infection (Laval et al., 2019). In addition to our discovery that pUL56 modulates TLR2 surface levels in infected cells, additional HSV-1 proteins have also been shown to inhibit TLR2 activity, including ICP0 (van Lint et al., 2010) and pUS3 (Sen et al., 2013). How the modulation of TLR2 by pUL56 and other viral proteins differentially affects the pathogenesis of herpesvirus infections awaits further study. Interestingly, GOPC may be a common target for modulation by viruses: human papillomavirus type 16 E6 protein was shown to bind GOPC and mediate its degradation through the host E3 ubiquitin ligase E6AP (Jeong et al., 2007). Unlike pUL56, E6 binds to the PDZ domain of GOPC through a PDZ-binding motif. In addition, the classical swine fever virus NS2 protein bound GOPC in a yeast two-hybrid screen (Kang et al., 2012), although it has not yet been determined whether GOPC is degraded during infection with this virus.

The pUL56 homologs from equine herpesvirus type 1 (EHV-1) and type 4 (EHV-4) share only 20% identity with HSV-1 pUL56, yet both are type II transmembrane proteins that possess multiple PPXY motifs and have few or no cytoplasmic lysine residues. Interestingly, both EHV-1 and EHV-4 have been shown to downregulate MHC-I from the surface of infected cells in a pUL56-dependent fashion (Ma et al., 2012; Said et al., 2012). Similarly, U24 from human herpesvirus 6A (HHV-6A) is a tail-anchored (type II) membrane protein containing a PPXY motif and has been shown to downregulate the T cell receptor (Koshizuka et al., 2018; Sullivan and Coscoy, 2008). Furthermore, HCMV UL42 has been shown to bind and stimulate ubiquitin-mediated degradation of ITCH (Koshizuka et al., 2016). It therefore seems likely that recruitment of NEDD4 family ubiquitin ligases by PPXY-motif-containing virally encoded type II transmembrane proteins is a conserved mechanism among herpesviruses to modulate membrane trafficking pathways in infected host cells.

In conclusion, our data provide extensive resources for understanding HSV interactions with host cells. Importantly, we identified that pUL56 targets GOPC for proteasomal degradation, thereby removing immune signaling molecules from the plasma membrane. This represents an elegant and efficient mechanism by which HSV-1 can remodel the surface of infected cells. The degradation of GOPC by other viruses may represent a common mechanism to modulate the cell surface of infected cells to evade host immune surveillance.

## STAR★Methods

### Key Resources Table

**Table:** 

REAGENT or RESOURCE	SOURCE	IDENTIFIER
**Antibodies**		

Rabbit monoclonal anti-GOPC (clone EPR4080(2))	Abcam	Cat#ab133472; RRID: AB_11156985
Mouse monoclonal anti-DNA PKcs (clone G4)	Santa Cruz Biotechnology	Cat#sc-5282; RRID: AB_2172848
Mouse monoclonal anti-IFI16 (clone 1G7)	Santa Cruz Biotechnology	Cat#sc-8023; RRID: AB_627775
Rabbit polyclonal anti-SETX	Stephen West, The Francis Crick Institute (Yüce and West, 2013)	OY7
Mouse monoclonal anti-ITCH (clone G-11)	Santa Cruz Biotechnology	Cat#sc-28367; RRID: AB_667798
Mouse monoclonal anti-GAPDH (clone 6C5)	ThermoFisher Scientific	Cat#AM4300; RRID: AB_2536381
Mouse monoclonal anti-Actin (clone AC-40)	Abcam	Cat#ab11003; RRID: AB_297660
Rat monoclonal anti-tubulin (clone YL1/2)	Abcam	Cat#ab6160; RRID: AB_305328
Mouse monoclonal anti-TLR2 (clone QA16A01)	BioLegend	Cat#153003; RRID: AB_2728203
Sheep polyclonal anti-TGN46	BioRad	Cat#AHP500G; RRID: AB_323104
Rabbit polyclonal anti-GFP	Sigma-Aldrich	Cat#G1544; RRID: AB_439690
Mouse monoclonal anti-c-Myc tag (clone 9E10)	Sigma-Aldrich	Cat#M4439; RRID: AB_439694
Mouse monoclonal anti-HA tag (HA.11 clone 16B12)	Covance	Cat#MMS-101R; RRID: AB_291262
Mouse monoclonal anti-FLAG tag (clone M2)	Sigma-Aldrich	Cat#F1804; RRID: AB_262044
Mouse monoclonal anti-HSV gD (clone LP2)	Tony Minson, University of Cambridge (Minson et al., 1986)	LP2
Mouse monoclonal anti-HSV VP16 (clone LP1)	Abcam	Cat#ab110226; RRID: AB_10863640
Mouse monoclonal anti-HSV ICP0 (clone 11060)	Chris Boutell, MRC-University of Glasgow Centre for Virus Research (Everett et al., 1993)	11060
Rabbit polyclonal anti-HSV-1 Vhs	Bernard Roizman, University of Chicago (Taddeo et al., 2006)	N/A
Rabbit polyclonal anti-HSV-1 pUL56	This paper	N/A
Donkey anti-Mouse IgG (H+L) Highly Cross-Adsorbed Secondary Antibody, Alexa Fluor 488	ThermoFisher Scientific	Cat#A-21202; RRID: AB_141607
Donkey anti-Rabbit IgG (H+L) Highly Cross-Adsorbed Secondary Antibody, Alexa Fluor 488	ThermoFisher Scientific	Cat#A-21206; RRID: AB_2535792
Donkey anti-Mouse IgG (H+L) Highly Cross-Adsorbed Secondary Antibody, Alexa Fluor 568	ThermoFisher Scientific	Cat#A10037;RRID: AB_2534013
Donkey anti-Rabbit IgG (H+L) Highly Cross-Adsorbed Secondary Antibody, Alexa Fluor 568	ThermoFisher Scientific	Cat#A10042; RRID: AB_2534017
Donkey anti-Sheep IgG (H+L) Highly Cross-Adsorbed Secondary Antibody, Alexa Fluor 647	ThermoFisher Scientific	Cat#A-21448; RRID: AB_2535865
IRDye® 680LT Goat anti-Mouse IgG (H + L)	Li-Cor	Cat#926-68020; RRID: AB_10706161
IRDye® 800CW Donkey anti-Rabbit IgG (H + L)	Li-Cor	Cat#926-32213; RRID: AB_621848
IRDye® 680LT Donkey anti-Rabbit IgG (H + L)	Li-Cor	Cat#926-68023; RRID: AB_10954442
IRDye® 800CW Goat anti-Mouse IgG (H + L)	Li-Cor	Cat#926-32210 RRID:AB_621842
Goat anti-Mouse HRP conjugated	CiteAb	Cat#P0447; RRID: AB_2617137

**Bacterial and Virus Strains**		

HSV-1 KOS BAC	David Leib, Geisel School of Medicine at Dartmouth, USA (Gierasch et al., 2006)	N/A
HSV-1 S17	Stacey Efstathiou, University of Cambridge	N/A
HSV-1 SC16	Tony Minson, University of Cambridge	N/A
HSV-2 333	Stacey Efstathiou, University of Cambridge	N/A
HSV-1 ΔUL56	This paper	N/A
HSV-1 pUL56-AAXA	This paper	N/A
HSV-1 ΔICP0	This paper	N/A
HSV-1 Δvhs	Zenner et al., 2013	N/A
BL21(DE3)pLysS *E. coli* cells	ThermoFisher Scientific	Cat#C606010
T7 Express lysY/Iq *E. coli*	New England Biolabs	Cat#C3013

**Chemicals, Peptides, and Recombinant Proteins**		

SILAC medium	Life Technologies	Cat#A33822
Peptide NH_2_-CTSSGEGEASERGRSR-CONH_2_	Eurogentech	N/A
Peptide Ac-AARGSSDHAPYRRQGC-CONH_2_	Eurogentec	N/A
SulfoLink Coupling Resin	ThermoFisher Scientific	Cat#20401
LysC protease, MS-grade	Wako	Cat#125-02543
Trypsin protease, MS-grade	Pierce	Cat#90058
Sep-Pak tC18 Vac Cartridge	Waters	Cat#WAT054960
Tandem mass tag (TMT) 10-plex isobaric reagents	Thermo Fisher Scientific	Cat#90110
TMT 16-plex isobaric reagents	Thermo Fisher Scientific	Cat#A44522
LC-MS grade Acetonitrile	Merck	Cat#1.00029.2500
Acetonitrile, Extra Dry	Acros Organics	Cat#AC364311000
Formic acid	Thermo Fisher	Cat#85178
Hydroxylamine	Sigma-Aldrich	Cat#438227
Aminooxy-biotin	Biotium	Cat#90113
Aniline	Sigma-Aldrich	Cat#242284
Triton X-100, high purity	ThermoFisher Scientific	Cat#28313
cOmplete, EDTA-free Protease Inhibitor Cocktail	Roche	Cat#11836153001
Iodoacetamide	Sigma-Aldrich	Cat#I1149-5G
Streptavidin agarose beads	ThermoFisher Scientific	Cat#20365
Kinetix Evo C18 column	Phenomenex	Cat#00F-4726-AN
PolySulfethyl A bulk material	Nest group	Cat#BMSE2003
Acclaim PepMap 100 C18 HPLC column	ThermoFisher Scientific	Cat#160454
Acclaim PepMap RSLC C18 column	ThermoFisher Scientific	Cat#164540
Acclaim PepMap RSLC C18 column	ThermoFisher Scientific	Cat#164536
TransIT®-LT1	Mirus	Cat#MIR2306
Lipofectamine 2000	ThermoFisher Scientific	Cat#11668019
ImmPACT DAB Peroxidase (HRP) Substrate	Vector Laboratories Ltd	Cat#SK-4105
ProLong Gold Antifade Mountant with DAPI	ThermoFisher Scientific	Cat#P36931
IgG from human serum	Sigma-Aldrich	Cat#I4506
Fetal Bovine Serum	PAN Biotech UK Ltd	Cat#P30-19375
Accutase	Sigma-Aldrich	Cat#A6964
EDTA-free Protease Inhibitor Cocktail	Sigma-Aldrich	Cat#P8849
Benzonase Nuclease	Sigma-Aldrich	Cat#E1014
GFP-Trap A beads	ChromoTek	Cat#gta-10
Myc-Trap beads	ChromoTek	Cat#yta-10
NiNTA agarose	QIAGEN	Cat#30230
Glutathione Sepharose 4B	GE Healthcare	Cat#17075604
Glutathione magnetic beads	ThermoFisher Scientific	Cat#11824131
InstantBlue Coomassie stain	Expedion	Cat#IST1L
MG132	Calbiochem	Cat#474790
DMSO	Sigma-Aldrich	Cat#D8418
N-ethylmaleimide	Sigma-Aldrich	Cat#E3876
Electron microscopy-grade formaldehyde	Polysciences	Cat#04018-1
Mowiol 4-88	Merck	Cat#475904
DAPI	Sigma-Aldrich	Cat#D8417

**Critical Commercial Assays**		

Micro BCA Protein Assay	ThermoFisher Scientific	Cat#23235
BCA Assay	ThermoFisher Scientific	Cat#23225

**Deposited Data**		

Mass spectrometry data	This paper. Deposited on PRIDE Archive (https://www.ebi.ac.uk/pride/archive)	http://www.ebi.ac.uk/pride/archive/projects/PXD021351

**Experimental Models: Cell Lines**		

Vero	ATCC	CRL-1586
HaCaT	Boukamp et al., 1988	N/A
HFF hTERT	McSharry et al., 2001	N/A
HEK293T	ATCC	CRL-3216
U2-OS	ATCC	HTB-96

**Oligonucleotides**		

COL581: Forward primer for deletion of pUL56 by Red recombination: CGACGCGGGTCTATGGAGCGCGGGG AACGCGTTTGCTGATTAGTAATGAATTC ACGATAGCTTGTCTGGTAGGaggatgacgacgataagtaggg	This paper	N/A
COL582: Reverse primer for deletion of pUL56 by Red recombination: ACGACAAACGGCCCCT CGTTCCTACCAGACAAGCTATCGTGAATTCATT ACTAATCAGCAAACGCGTTCCCCGcaaccaat taaccaattctgattag	This paper	N/A
COL579: Forward primer for deletion of ICP0 by Red recombination: GACCCCCATGGAGCCCCGCCCCGG AGCGAGTACCCGCCGGTAGTAATGAATTCCCCAGC GCGAGGTGAGGGGCAGGATGACGACGATAAGTAGGG	This paper	N/A
COL580: Reverse primer for deletion of ICP0 by Red recombination: CGCCCCAGACATGGCGCCCGGCC CCTCACCTCGCGCTGGGGAATTCATTACTACCGGC GGGTACTCGCTCCGGcaaccaattaaccaattctgattag	This paper	N/A
CRISPR GOPC gRNA 1: GGAACATGGATACCCCGCCA	This paper	N/A
CRISPR GOPC gRNA 2: GAGAGATCGATCCAGACCAAG	This paper	N/A

**Recombinant DNA**		

Plasmid: GFP-pUL56(1-234)	This paper	N/A
Plasmid: GFP-pUL56(1-207)	This paper	N/A
Plasmid: GFP-pUL56(1-157)	This paper	N/A
Plasmid: GFP-pUL56(1-104)	This paper	N/A
Plasmid: GFP-pUL56(1-74)	This paper	N/A
Plasmid: pUL56(1-207)-GFP	This paper	N/A
Plasmid: GFP-pUL56-AAXA	This paper	N/A
Plasmid: YFP-NEDD4-WW	Juan Martin-Serrano, King’s College London; (Martin-Serrano et al., 2005)	N/A
Plasmid: pF5K myc-GOPC	This paper	N/A
Plasmid: HA-Ub (pMT123)	Paul Lehner, University of Cambridge	N/A
Plasmid: GST-UL56(1-207)-His6	This paper	N/A
Plasmid: His-GOPC(1-454)	This paper	N/A
Plasmid: His-GOPC(1-362)	This paper	N/A
Plasmid: His-GOPC(27-362)	This paper	N/A
Plasmid: His-GOPC(276-362)	This paper	N/A
Plasmid: His-GOPC(27-236)	This paper	N/A
Plasmid: FLAG-tagged TLR2	Nick Gay, University of Cambridge	N/A
Plasmid: pEGFP-N1	Clontech	Cat#6085-1
Plasmid: pSpCas9(BB)-2A-Puro (PX459) V2	Feng Zhang, Broad Institute (Ran et al., 2013)	Addgene plasmid #62988

**Software and Algorithms**		

“MassPike,” a Sequest-based software pipeline for quantitative proteomics	Professor Steven Gygi’s lab, Harvard Medical School, Boston, USA	N/A
MaxQuant v. 1.5.7.4 and 1.5.8.3	(Cox and Mann, 2008)	https://www.maxquant.org/maxquant/
Perseus v. 1.5.1.6 and v. 1.6.1.2	(Tyanova et al., 2016)	https://www.maxquant.org/perseus/
Cluster 3.0	(de Hoon et al., 2004)	http://bonsai.hgc.jp/∼mdehoon/software/cluster/software.htm
Java Treeview	(Saldanha, 2004)	http://jtreeview.sourceforge.net/
XLSTAT v. 18.06	Addinsoft	https://www.xlstat.com/en/
Image Studio v. 5.2	LI-COR	https://www.licor.com/bio/image-studio/
DAVID v. 6.8	(Huang et al., 2009)	https://david.ncifcrf.gov/
Image-Pro Plus	Media Cybernetic	https://www.mediacy.com/imagepro
Image-J	Research Services Branch	https://imagej.nih.gov/ij/
Flowing Software v. 2.5.1	Perttu Terho, Turku Centre for Biotechnology	http://flowingsoftware.btk.fi/

**Other**		

Unprocessed peptide data files for Figures 1, 3, 5, 6, and 7	This paper. Deposited on Mendeley Data (https://data.mendeley.com)	https://doi.org/10.17632/g5sf93zwtf.3

### Resource Availability

#### Lead Contact

Further information and requests for resources and reagents should be directed to and will be fulfilled by the Lead Contact, Colin M. Crump (cmc56@cam.ac.uk).

#### Materials Availability

Newly generated materials associated with this study, including plasmids, viruses, cell lines and antibodies, are available on request from the Lead Contact.

#### Data and Code Availability

The mass spectrometry proteomics data have been deposited to the ProteomeXchange Consortium via the PRIDE (Perez-Riverol et al., 2019) partner repository with the dataset identifier PXD021351 (http://www.ebi.ac.uk/pride/archive/projects/PXD021351).

Unprocessed peptide data files for Figures 1, 3, 5, 6, and 7 are available at https://data.mendeley.com/ with the digital object identifier https://doi.org/10.17632/g5sf93zwtf.3.

### Experimental Model and Subject Details

#### Viral and bacterial strains

See Key Resources Table for details of all virus and bacteria strains used in this research.

#### Cell lines

The following mammalian cell lines were used in this work: HaCaT cells: human keratinocyte cell line, spontaneously immortalized, male (Boukamp et al., 1988); HFF hTERT cells: human foreskin fibroblast cell line, telomerase immortalized, male (McSharry et al., 2001); Vero cells: African green monkey kidney cell line, spontaneously immortalized, female (ATCC, CRL-1586); HEK293T cells: human embryonic kidney cell line, Adenovirus 5 and SV40 transformed, female (ATCC, CRL-3216); U2-OS cells: human osteosarcoma cell line, cancer cell line, female (ATCC, HTB-96). All cell lines were maintained in Dulbecco’s Modified Eagle’s Medium (DMEM) supplemented with 10% (v/v) heat-inactivated 0.2 μm sterile filtered fetal bovine serum (FBS; PAN Biotech UK Ltd), 2 mM L-glutamine, 100 U/mL penicillin, and 100 μg/mL streptomycin and cells were grown at 37°C in a humidified 5% CO_2_ atmosphere. For stable isotope labeling of amino acids in cell culture (SILAC) experiments, HEK293T or HaCaT cells were grown in SILAC medium (high glucose DMEM lacking arginine and lysine, Life Technologies) supplemented with 10% (v/v) dialyzed heat-inactivated 0.2 μm sterile filtered fetal bovine serum (10 kDa cutoff), 2 mM glutamine, 100 U/mL penicillin and 100 μg/mL streptomycin. Media were supplemented with 84 mg/L arginine (light, unlabelled; medium, Arg6 (^13^C6); heavy, Arg10 (^13^C6, ^15^N4)) and 146 mg/L lysine (light, unlabelled; medium, Lys4 (^2^H4); heavy, Lys8 (^13^C6, ^15^N2)). Cells were maintained in SILAC media for at least five passages before use to ensure complete labeling.

#### Viruses

All HSV-1 strain KOS viruses were reconstituted from a bacterial artificial chromosome (Gierasch et al., 2006). The deletion mutants were generated by inserting three tandem stop codons in frame using the two-step Red recombination method (Tischer et al., 2010). For ΔUL56 this is after residue 21, for ΔICP0 this is after residue 11, and for Δvhs this is after residue 45 (Zenner et al., 2013). To generate a mutant virus expressing pUL56 that lacks all three PPXY motifs, site-directed mutagenesis was first used to generate pUL56 where all three PPXY motifs had been mutated to AAXA. A plasmid containing pUL56-AAXA and an I-SceI/KanR selection cassette was then used to generate a recombinant HSV-1 strain KOS using the two-step Red recombination method, and the presence of the correct mutated sites in the reconstituted virus genome confirmed by sequencing the UL56 region. HSV-1 S17 and HSV-2 333 were from S. Efstathiou (University of Cambridge), and HSV-1 SC16 was from T. Minson (University of Cambridge). Crude stocks were generated by infecting Vero cells at MOI of 0.01. After 3 days, the cells were scraped and isolated by centrifugation at 900 × g for 5 min. They were resuspended in 1 mL of complete media per T150 used and freeze/thawed thrice at −70°C before being aliquoted, titered on Vero cell monolayers, and stored at −70°C until required.

### Method Details

#### Gradient purification of HSV-1

HaCaT cells were seeded and infected with crude virus stocks at MOI of 0.1. After 2 days, the cells were scraped and the cell debris was removed by centrifugation at 900 × g for 5 min. The supernatant was ultracentrifuged at 24,000 × g for 1.5 h, and the pellet was resuspended in 1% FBS in PBS on ice overnight. This solution was overlaid on a 5%–15% (w/v) continuous Ficoll in PBS gradient and ultracentrifuged at 17,500 × g for 1.5 h. The virion band was isolated via side-puncture. This solution was diluted 10-fold in PBS, and the virus was pelleted by ultracentrifugation at 49,000 × g for 2 h. The pellet was resuspended in PBS on ice overnight. This solution was aliquoted, titered on Vero cell monolayers, and stored at −70°C until required.

#### Antibodies

See Key Resources Table for details of all primary and secondary antibodies used in this research.

An antibody against pUL56 was generated by commercial immunization of a rabbit using two peptides (peptide 1: NH_2_-CTSSGEGEASERGRSR-CONH_2_; peptide 2: Ac-AARGSSDHAPYRRQGC-CONH_2_) coupled to keyhole limpet hemocyanin (Eurogentec). An affinity purification column was generated by adding 0.96 mg of purified peptide 1 dissolved in coupling buffer (250 mM Tris pH 8.5, 25 mM EDTA) to 0.4 mL of SulfoLink resin (ThermoFisher) equilibrated in the same buffer. The resin was incubated with the peptide for two hours at 20°C with regular mixing, washed with 1.2 mL of coupling buffer and then blocked using 50 mM cysteine in coupling buffer at 20°C for 90 minutes with regular mixing. The resin was subsequently washed twice with 1 mL of 1 M NaCl, followed by another two washes with 5 mL of PBS. The immune serum was mixed with an equal volume of PBS and incubated with the peptide-coupled resin for 20 h at 4°C. The affinity-purified antibody was eluted in fractions using 100 mM glycine pH 2.5 into tubes containing 10 × neutralization buffer (1M Tris pH 8.5, 2 M NaCl). Specificity of the antibody for use in immunoblots was tested by probing against cell lysates where pUL56 was absent or overexpressed, against lysates of cells infected with HSV-1 WT or ΔUL56, and against the GST-tagged purified recombinant protein (see below). BSA was added to the antibody for stabilization (final concentration 1 mg/mL) and the antibody was stored as a 50% (v/v) glycerol stock at −20°C.

#### Infection

Cell monolayers were infected with HSV-1 at the specified MOI diluted in complete media. For experiments to be analyzed by mass spectrometry, gradient-purified virus stocks were used. Otherwise, the infection was performed with crude virus stocks generated as described above. After adsorption for 1 h at 37°C with 5% CO_2_ and rocking every 15 min, the appropriate media was added to the well and this was designated 0 hpi. Infected cells were incubated at 37°C in a humidified 5% CO_2_ atmosphere until harvest.

#### Whole cell lysate sample preparation for quantitative temporal viromics

HaCaT cells were seeded into 6-well plates and infected in parallel at the specified MOI with gradient purified virus. At each indicated time point, cells were washed twice with PBS, and 250 μL lysis buffer was added (6M guanidine, 50 mM HEPES pH 8.5). Cell lifters (Corning) were used to scrape cells in lysis buffer, which was removed to a microcentrifuge tube, vortexed extensively, and then sonicated and snap frozen in liquid nitrogen. After harvest, samples were stored at −70°C until all time points were harvested. Samples were thawed and cell debris was removed by centrifuging at 21,000 × g for 10 min twice. Dithiothreitol (DTT) was added to a final concentration of 5 mM and samples were incubated for 20 min. Cysteines were alkylated with 14 mM iodoacetamide and incubated 20 min at room temperature in the dark. Excess iodoacetamide was quenched with DTT for 15 mins. Samples were diluted with 200 mM HEPES pH 8.5 to 1.5 M guanidine, followed by digestion at room temperature for 3 h with LysC protease (Wako) at a 1:100 protease-to-protein ratio. Samples were further diluted with 200 mM HEPES pH 8.5 to 0.5 M guanidine. Trypsin (Pierce) was then added at a 1:100 protease-to-protein ratio followed by overnight incubation at 37°C. The reaction was quenched with 5% (v/v) formic acid (FA; Thermo-Fisher), then centrifuged at 21,000 × g for 10 min to remove undigested protein. Peptides were subjected to C18 solid-phase extraction (SPE; Sep-Pak, Waters) and vacuum-centrifuged to near-dryness.

#### Peptide labeling with tandem mass tags for whole cell experiments

In preparation for TMT labeling, desalted peptides were dissolved in 200 mM HEPES pH 8.5. Peptide concentration was measured by microBCA (Pierce), and > 25 μg of peptide were labeled with TMT reagent. TMT reagents (0.8 mg) were dissolved in 43 μL anhydrous acetonitrile (Acros Organics) and 5 μL was added to the peptides at a final anhydrous acetonitrile concentration of 30% (v/v). Sample labeling was as indicated in Tables S1 and S4. Following incubation at room temperature for 1 h, the reaction was quenched with hydroxylamine (Sigma-Aldrich) to a final concentration of 0.5%. TMT-labeled samples were combined at a 1:1:1:1:1:1:1:1:1:1 ratio. The sample was vacuum-centrifuged to near dryness and subjected to C18 SPE (Sep-Pak, Waters). An unfractionated sample was analyzed initially to ensure similar peptide loading across each TMT channel, to avoid the need for excessive (> 2-fold) electronic normalization. Samples were combined according to the correction factors from the unfractionated analysis and subjected to C18 SPE (Sep-Pak, Waters) and vacuum-centrifuged to near-dryness. The dried pellet was resuspended in 200 mM ammonium formate pH 10 and subjected to high pH reversed-phase (HpRP) fractionation is as described below.

#### Sample preparation for plasma membrane profiling

For the SILAC-based plasma membrane profiling experiment (Figure 6), SILAC labeled HaCaT cells (as described above) were grown in 15 cm dishes and infected with gradient purified HSV-1 WT or HSV-1 ΔUL56 or mock infected in complete media at MOI 10. Plasma membrane profiling was performed as described previously with minor modifications (Weekes et al., 2010). At 6 hpi cells were washed twice in ice-cold PBS. Surface sialic acid residues were oxidized and biotinylated for 30 min on ice in the dark using an oxidation/biotinylation mix comprising 1 mM sodium meta-periodate, 100 mM aminooxy-biotin (Biotium Inc., Hayward, CA) and 10 mM aniline (Sigma-Aldrich) in ice-cold PBS pH 6.7. The reaction was quenched by glycerol to 1 mM final concentration and cells were washed twice in ice-cold PBS. Biotinylated cells were scraped into lysis buffer (1% Triton X-100 (high purity, ThermoFisher Scientific), 150 mM NaCl, 1 × protease inhibitor (complete, without EDTA (Roche)), 5 mM iodoacetamide (Sigma-Aldrich), and 10 mM Tris-HCl pH 7.6) then incubated on ice for 30 mins. Nuclei were removed by centrifugation at 4°C. Biotinylated glycoproteins were enriched by incubation for 2 h at 4°C with high affinity streptavidin agarose beads (ThermoFisher Scientific). Extensive washing was performed on a vacuum manifold, using lysis buffer, then PBS/0.5% (w/v) SDS. Beads were incubated for 20 min at RT with PBS/0.5% (w/v) SDS/100 mM DTT. Further washing was performed with UC buffer (6 M urea, 100 mM Tris-HCl pH 8.5), followed by alkylation for 20 min at room temperature with UC buffer containing 50 mM iodoacetamide. Beads were washed using UC buffer, 5 M NaCl, then water. Captured protein was digested on-bead with trypsin in 100 mM HEPES pH 8.5 for 3 h. Tryptic peptides were collected and fractionated by tip-based SCX strong cation exchange (described below), generating six fractions for MS analysis.

For the TMT-based GOPC knockout plasma membrane experiment (Figure 7), cells were seeded into 15 cm dishes and plasma membrane profiling was performed as described above the following day. Tryptic peptides were labeled with TMT as described for whole cell experiments, except 10 μL TMT reagent was added to the entire peptide sample and the reaction was not immediately quenched after labeling. To assess TMT incorporation and to ensure equal peptide loading, 10% of each labeled sample was quenched and combined for initial analysis. If incorporation was below 95%, samples were re-labeled with a further 10 μL TMT reagent prior to quenching. Six fractions were generated by HpRp fractionation, as described below.

#### Offline HpRP fractionation for TMT-based proteomics

TMT-labeled tryptic peptides were subjected to HpRP fractionation using an Ultimate 3000 RSLC UHPLC system (Thermo Fisher Scientific) equipped with a 2.1 mm internal diameter (ID) x 15 cm long, 1.7 μm particle Kinetix Evo C18 column (Phenomenex). Mobile phase consisted of A: 3% (v/v) acetonitrile (MeCN, Merck), B: MeCN and C: 200 mM ammonium formate pH 10. Isocratic conditions were 90% A / 10% C, and C was maintained at 10% throughout the gradient elution. Separations were conducted at 45°C. Samples were loaded at 200 μL/min for 5 min. The flow rate was then increased to 400 μL/min over 5 min, after which the gradient elution proceed as follows: 0%–19% B over 10 min, 19%–34% B over 14.25 min, 34%–50% B over 8.75 min, followed by a 10 min wash at 90% B. UV absorbance was monitored at 280 nm and 15 s fractions were collected into 96-well microplates using the integrated fraction collector. Fractions were recombined orthogonally in a checkerboard fashion, combining alternate wells from each column of the plate into a single fraction, and commencing combination of adjacent fractions in alternating rows. Wells prior to the start or after the stop of elution of peptide-rich fractions, as identified from the UV trace, were excluded. This yielded two sets of 12 combined fractions, A and B, which were dried in a vacuum centrifuge and resuspended in 10 μL MS solvent (4% (v/v) MeCN / 5% (v/v) FA) prior to LC-MS3. For the time course experiment (Figure 1A) and ΔUL56/wild-type HSV-1 whole cell lysate experiment (Figure 5D), 12 set ‘A’ fractions were used for MS analysis. For the GOPC knockout plasma membrane profiling experiment, 6 combined fractions were instead analyzed. These were generated by recombining all wells from sets of two adjacent columns in the plate (i.e., columns A+B, C+D, E+F etc).

#### Offline Tip-Based Strong Cation Exchange SCX Fractionation

Our previously described protocol for solid-phase extraction-based SCX peptide fractionation was modified for small peptide amounts (Dephoure and Gygi, 2011). Briefly, 10 mg of PolySulfethyl A bulk material (Nest Group Inc.) was loaded on to a fritted 200 μL tip in 100% Methanol using a vacuum manifold. SCX material was conditioned slowly with 1 mL SCX buffer A (7M KH_2_PO_4_, pH 2.65, 30% (v/v) MeCN), then 0.5 mL SCX buffer B (7 mM KH_2_PO_4_, pH 2.65, 350 mM KCl, 30% (v/v) MeCN) then 2 mL SCX buffer A. Dried peptides were resuspended in 500 mL SCX buffer A and added to the tip at a flow rate of ~150 mL/min, followed by a 150 mL wash with SCX buffer A. Fractions were eluted in 150 μL buffer at increasing K^+^ concentrations (10, 25, 40, 60, 90, 150 mM KCl), vacuum-centrifuged to near dryness, then desalted using StageTips and vacuum-centrifuged to complete dryness and resuspended in 10 μL MS solvent (4% (v/v) MeCN / 5% (v/v) FA) prior to LC-MS3.

#### LC-MS/MS/MS for TMT-based proteomics

Mass spectrometry data was acquired using an Orbitrap Lumos (Thermo Fisher Scientific, San Jose, CA). An Ultimate 3000 RSLC nano UHPLC equipped with a 300 μm ID x 5 mm Acclaim PepMap μ-Precolumn (Thermo Fisher Scientific) and a 75 μm ID x 50 cm 2.1 μm particle Acclaim PepMap RSLC analytical column was used. Loading solvent was 0.1% FA, analytical solvent A: 0.1% FA and B: 80% (v/v) MeCN + 0.1% FA. All separations were carried out at 55°C. Samples were loaded at 5 μL/min for 5 min in loading solvent before beginning the analytical gradient. For whole cell lysate experiments, the following gradient was used: 3%–7% B over 3 min, 7%–37% B over 173 min, followed by a 4-min wash at 95% B and equilibration at 3% B for 15 min. For plasma membrane profiling experiments, the following gradient was used: 3%–7% B over 3 min, 7%–37% B over 116 min, followed by a 4-min wash at 95% B and equilibration at 3% B for 15 min. Each analysis used a MultiNotch MS3-based TMT method (McAlister et al., 2012, 2014). The following settings were used: MS1: 380-1500 Th, 120,000 Resolution, 2 × 10^5^ automatic gain control (AGC) target, 50 ms maximum injection time. MS2: Quadrupole isolation at an isolation width of m/z 0.7, CID fragmentation (normalized collision energy (NCE) 35) with ion trap scanning in turbo mode from m/z 120, 1.5 × 10^4^ AGC target, 120 ms maximum injection time. MS3: In Synchronous Precursor Selection mode the top 6 MS2 ions were selected for HCD fragmentation (NCE 65) and scanned in the Orbitrap at 60,000 resolution with an AGC target of 1 × 10^5^ and a maximum accumulation time of 150 ms. Ions were not accumulated for all parallelizable time. The entire MS/MS/MS cycle had a target time of 3 s. Dynamic exclusion was set to ± 10 ppm for 70 s. MS2 fragmentation was trigged on precursors 5 × 10^3^ counts and above.

#### TMT Data analysis

In the following description, we list the first report in the literature for each relevant algorithm. Mass spectra were processed using a Sequest-based software pipeline for quantitative proteomics, “MassPike,” through a collaborative arrangement with Professor Steve Gygi’s laboratory at Harvard Medical School. MS spectra were converted to mzxml using an extractor built upon Thermo Fisher’s RAW File Reader library (version 4.0.26). In this extractor, the standard mzxml format has been augmented with additional custom fields that are specific to ion trap and Orbitrap mass spectrometry and essential for TMT quantitation. These additional fields include ion injection times for each scan, Fourier Transform-derived baseline and noise values calculated for every Orbitrap scan, isolation widths for each scan type, scan event numbers, and elapsed scan times. This software is a component of the MassPike software platform and is licensed by Harvard Medical School.

A combined database was constructed from (a) the human UniProt database (26th January, 2017), (b) HSV-1 strain KOS (GenBank entry JQ673480.1, manually updated with a single amino acid polymorphism in the ICP4 sequence identified in the KOS BAC strain used for virus generation), (c) common contaminants such as porcine trypsin and endoproteinase LysC. The combined database was concatenated with a reverse database composed of all protein sequences in reversed order. Searches were performed using a 20 ppm precursor ion tolerance (Haas et al., 2006). Product ion tolerance was set to 0.03 Th. TMT tags on lysine residues and peptide N termini (229.162932 Da) and carbamidomethylation of cysteine residues (57.02146 Da) were set as static modifications, while oxidation of methionine residues (15.99492 Da) was set as a variable modification.

To control the fraction of erroneous protein identifications, a target-decoy strategy was employed (Elias and Gygi, 2010). Peptide spectral matches (PSMs) were filtered to an initial peptide-level false discovery rate (FDR) of 1% with subsequent filtering to attain a final protein-level FDR of 1% (Kim et al., 2011; Wu et al., 2011). PSM filtering was performed using a linear discriminant analysis (Huttlin et al., 2010). This distinguishes correct from incorrect peptide IDs in a manner analogous to the widely used Percolator algorithm (Käll et al., 2007), though employing a distinct machine learning algorithm. The following parameters were considered: XCorr, ΔCn, missed cleavages, peptide length, charge state, and precursor mass accuracy. Protein assembly was guided by principles of parsimony to produce the smallest set of proteins necessary to account for all observed peptides (Huttlin et al., 2010). Where all PSMs from a given HSV-1 protein could be explained either by a canonical gene or non-canonical ORF, the canonical gene was picked in preference.

In three cases, PSMs assigned to non-canonical ORFs (6FT-ORFs) were a mixture of peptides from the canonical protein and the 6FT-ORF. This most commonly occurred where the 6FT-ORF was a 5′-terminal extension of the canonical protein (thus meaning that the smallest set of proteins necessary to account for all observed peptides included the 6FT-ORFs alone). In these cases, the peptides corresponding to the canonical protein were separated from those specific to the 6FT-ORF, generating two separate entries.

Proteins were quantified by summing TMT reporter ion counts across all matching peptide-spectral matches using ”MassPike,” as described (McAlister et al., 2012, 2014). Briefly, a 0.003 Th window around the theoretical m/z of each reporter ion (126, 127n, 127c, 128n, 128c, 129n, 129c, 130n, 130c, 131n, 131c) was scanned for ions, and the maximum intensity nearest to the theoretical m/z was used. The primary determinant of quantitation quality is the number of TMT reporter ions detected in each MS3 spectrum, which is directly proportional to the signal-to-noise (S:N) ratio observed for each ion (Makarov and Denisov, 2009). Conservatively, every individual peptide used for quantitation was required to contribute sufficient TMT reporter ions (minimum of ~1250 per spectrum) so that each on its own could be expected to provide a representative picture of relative protein abundance (McAlister et al., 2012). Additionally, an isolation specificity filter was employed to minimize peptide co-isolation (Ting et al., 2011). Peptide-spectral matches with poor quality MS3 spectra (more than 9 TMT channels missing and/or a combined S:N ratio of less than 250 across all TMT reporter ions) or no MS3 spectra at all were excluded from quantitation. Peptides meeting the stated criteria for reliable quantitation were then summed by parent protein, in effect weighting the contributions of individual peptides to the total protein signal based on their individual TMT reporter ion yields. Protein quantitation values were exported for further analysis in Excel (Microsoft).

For protein quantitation, reverse and contaminant proteins were removed, then each reporter ion channel was summed across all quantified proteins and normalized assuming equal protein loading across all channels. For further analysis and display in figures, fractional TMT signals were used (i.e., reporting the fraction of maximal signal observed for each protein in each TMT channel, rather than the absolute normalized signal intensity). This effectively corrected for differences in the numbers of peptides observed per protein. For TMT experiments, normalized S:N values are presented in Tables S1 and S4 (‘Data’ worksheet).

Significance B was used to estimate the probability that each ratio was significantly different to 1 (Cox and Mann, 2008). Values were calculated and corrected for multiple hypothesis testing using the method of Benjamini-Hochberg in Perseus version 1.5.1.6 (Cox and Mann, 2008). A corrected p value < 0.05 was considered statistically significant. Hierarchical centroid clustering based on uncentered Pearson correlation of data normalized by comparing the signal:noise values to the average mock-infection were performed using Cluster 3.0 (Stanford University) (de Hoon et al., 2004), and visualized using Java Treeview (http://jtreeview.sourceforge.net) (Saldanha, 2004). For analysis of temporal classes, viral protein expression was normalized and subjected to K-means analysis using XLSTAT base (Addinsoft, version 18.06) and clustered with 1-15 classes.

#### LC-MS/MS and data analysis for SILAC-based plasma membrane experiments

Mass spectrometry data was acquired using an Orbitrap Lumos (Thermo Fisher Scientific, San Jose, CA). An Ultimate 3000 RSLC nano UHPLC equipped with a 300 μm ID x 5 mm Acclaim PepMap μ-Precolumn (Thermo Fisher Scientific) and a 75 μm ID x 50 cm 2.1 μm particle Acclaim PepMap RSLC analytical column was used. Loading solvent was 0.1% FA, analytical solvent A: 0.1% FA and B: 80% (v/v) MeCN + 0.1% FA. All separations were carried out at 55°C. Samples were loaded at 5 μL/min for 5 min in loading solvent before beginning the analytical gradient. The following gradient was used: 3%–7% B over 4 min, 7%–37% B over 116 min, followed by a 4-min wash at 95% B and equilibration at 3% B for 15 min. Each analysis used an MS2 DDA acquisition using the following settings: MS1: 375-1500 Th, 60,000 Resolution, 4 × 10^5^ automatic gain control (AGC) target, 50 ms maximum injection time. MS2: Quadrupole isolation at an isolation width of m/z 1.6, HCD fragmentation (normalized collision energy (NCE) 35) with ion trap scanning in rapid mode from m/z 110, 1 × 10^4^ AGC target, 35 ms maximum injection time.

The resulting spectra were processed in Maxquant 1.5.8.3 using medium (Arg6, Lys4) and heavy (Arg10, Lys8) labels. Data was searched against the human and HSV-1 strain KOS proteomes as used for TMT analysis (above). Carbamidomethyl (C) was set as a fixed modification, oxidation (M) and acetylation (protein N termini) set as variable modifications. Protein and peptide FDR were both set to 0.01, re-quantify was enabled and minimum ratio count was set to 2. Hierarchical centroid clustering based on uncentered Pearson correlation of the normalized ratios generated by MaxQuant was performed using Cluster 3.0 (Stanford University) (de Hoon et al., 2004) and visualized with Java Treeview (http://jtreeview.sourceforge.net) (Saldanha, 2004).

#### Immunoblot of cell lysates

Cells were seeded into 24-well plates and infected with crude virus stocks in complete media. Where indicated, cells were treated with 10 μM MG132 (Calbiochem) or an equivalent volume of carrier (DMSO). Cells were harvested at the specified time point by scraping into the media and centrifuging at 16,000 × g for 1 min. The cell pellet was resuspended in SDS loading buffer (50 mM Tris pH 6.8, 100 mM 2-mercaptoethanol, 2% (w/v) SDS, 10% (v/v) glycerol). Samples were immediately boiled in a water bath for 5 min. Lysate from 1 × 10^5^ cells was used for SDS-PAGE. Proteins were wet transferred onto 0.45 μm nitrocellulose membrane. After incubation with a primary antibody, secondary antibodies conjugated to an IRDye were used, and blots were visualized with an Odyssey CLx Imaging System (Li-Cor) using control software Image Studio v5.2.

#### Pathway analysis

The Database for Annotation, Visualization and Integrated Discovery (DAVID) version 6.8 was used to determine pathway enrichment (Huang et al., 2009). Proteins downregulated > 2-fold were searched against a background of all proteins quantified, using default settings.

#### Immunoprecipitation

Monolayers of HEK293T cells grown in 9 cm dishes (5 × 10^6^ cells/dish) were transfected using lipofectamine 2000 (Invitrogen) or TransIT-LT1 (Mirus) with expression plasmids. For identification of interaction partners and GOPC-binding domain analysis, plasmids expressing full length pUL56 or various truncation constructs of pUL56 as EGFP fusion proteins or EGFP alone were used. For analysis of tripartite complex formation plasmids expressing the WW domains of NEDD4 fused to EYFP (Martin-Serrano et al., 2005), myc-tagged GOPC and either wild-type pUL56 or pUL56-AAXA were used. For GOPC ubiquitination analysis, plasmids expressing the HA-tagged ubiquitin, myc-tagged GOPC and either wild-type pUL56 or pUL56-AAXA were used. For experiments with SILAC-labeled cells, the relevant labeled medium was used to prepare the transfection reagent. For protein interaction assays, cells were harvested 16-24 h post-transfection by scraping into the medium, pelleted (220 × g, 5 min, 4°C) and washed three times with cold PBS. Cells were then lysed at 4°C in 1 mL lysis buffer (10 mM Tris pH 7.5, 150 mM NaCl, 2 mM MgCl_2_, 0.5% Triton X-100, 1:100 diluted EDTA-free protease inhibitor cocktail (Sigma-Aldrich), 50 U/mL benzonase (Sigma-Aldrich)) for 45-90 min. For GOPC ubiquitination analysis, cells were treated with 10 μM MG132 at 30 h post-transfection and harvested 16 h later by scraping into PBS, pelleted (220 × g, 5 min, 4°C) and lysed in a modified lysis buffer (50 mM Tris pH 7.5, 150 mM NaCl, 1 mM EDTA, 1 mM EGTA, 1% Triton X-100, 1% sodium deoxycholate, 100mM N-ethylmaleimide, cOmplete protease inhibitor cocktail (Roche)). The cell lysate was clarified by centrifugation (20,000 × g, 10 min, 4°C), the supernatant transferred to fresh tubes, a BCA assay (Pierce) was performed to measure total protein concentration of clarified cell lysates, and samples were normalized (*input*).

GFP-Trap or Myc-Trap Agarose beads (ChromoTek, 20 μL per sample) were washed three times by dilution in 800 μL wash buffer (10 mM Tris pH 7.5, 150 mM NaCl, 2 mM MgCl_2_, 0.05% Triton X-100), centrifugation (2500 × g, 2 min, 4°C) to collect the beads and removal of the supernatant. Washed beads were incubated with the cleared lysate at 4°C on a rotating wheel for 45-70 min. The beads were collected by centrifugation and the supernatant (*unbound*) was removed. The beads were washed twice with 1 mL wash buffer, the supernatant was discarded, 45 μL of 2 × SDS-PAGE loading buffer was added per experiment and the was mixture boiled at 95°C for 10 min to elute bound proteins. Samples were centrifuged again to sediment the beads (20,000 × g, 2 min) and the supernatant (*bound*) was transferred to a fresh tube. Input, unbound and bound samples were separated by SDS-PAGE and analyzed by immunoblot. For mass spectroscopy analysis of SILAC samples, 8 μL of light-, medium- and heavy-labeled bound samples were mixed in a 1:1:1 ratio and frozen at −80°C until mass spectroscopy analysis.

#### Mass spectrometry of SILAC IP samples

Mass spectrometry analysis was performed by the proteomics facility of the University of Bristol (UK). Three biological repeats of each triple-labeled SILAC IP experiment were analyzed. Samples were run into precast SDS-PAGE gels for 5 minutes, the entire sample extracted from the gel as a single band, and then in-gel digested, reduced and alkylated using a ProGest automated digestion unit (Digilab). The resulting peptides were fractionated using an Ultimate 3000 nano-LC system in line with an Orbitrap Fusion Tribrid mass spectrometer (Thermo Scientific). In brief, peptides in 1% (v/v) FA were injected onto an Acclaim PepMap C18 nano-trap column (Thermo Scientific). After washing with 0.5% MeCN in 0.1% FA, peptides were resolved on a 250 mm × 75 μm Acclaim PepMap C18 reverse phase analytical column (Thermo Scientific) over a 150 min organic gradient using 7 gradient segments (1%–6% solvent B over 1 min, 6%–15% B over 58 min, 15%–32% B over 58 min, 32%–40% B over 5 min, 40%–90% B over 1 min, held at 90% B for 6 min and then reduced to 1% B over 1min) with a flow rate of 300 nL per minute. Solvent A was 0.1% FA and solvent B was aqueous 80% MeCN in 0.1% FA. Peptides were ionized by nano-electrospray ionization at 2.0 kV using a stainless steel emitter with an internal diameter of 30 μm (Thermo Scientific) and a capillary temperature of 275°C. All spectra were acquired using an Orbitrap Fusion Tribrid mass spectrometer controlled by Xcalibur 2.1 software (Thermo Scientific) and operated in data-dependent acquisition mode. FTMS1 spectra were collected at a resolution of 120,000 over a scan range (m/z) of 350-1550, with an automatic gain control (AGC) target of 300,000 and a max injection time of 100 ms. Precursors were filtered using an Intensity Range of 1 × 10^4^ to 1 × 10^20^ and according to charge state (to include charge states 2-6) and with monoisotopic precursor selection. Previously interrogated precursors were excluded using a dynamic window (40 s ± 10 ppm). The MS2 precursors were isolated with a quadrupole mass filter set to a width of 1.4 m/z. ITMS2 spectra were collected with an AGC target of 20,000, max injection time of 40 ms and CID collision energy of 35%.

The raw data files were processed using MaxQuant v. 1.5.7.4 (Cox and Mann, 2008). The in-built Andromeda search engine (Cox et al., 2011) was used to search against the human and HSV-1 strain KOS proteomes as used for TMT analysis (above). Trypsin/P digestion, standard modifications (oxidation, N-terminal acetylation) were selected as group-specific parameters and SILAC quantification was performed using light (Arg0, Lys0), medium (Arg6, Lys4) and heavy (Arg10, Lys8) labels. Re-quantification, razor protein FDR, and second peptide options were enabled for the processing. The quantified data were analyzed with Perseus v. 1.6.1.2 (Tyanova et al., 2016) using the normalized ratios obtained by MaxQuant. Proteins only identified by site or against the reverse database, as well as common experimental contaminants such as keratins (specified in the MaxQuant contaminants file), were removed and the experiments grouped by biological repeat. Only proteins identified in at least two of the three biological repeats were considered for analysis. A one-sample, two-sided t test with a threshold p value of 0.05 was performed on each group to identify significantly enriched proteins. Proteins with a log_2_ fold change greater than 1 and a p value smaller than 0.05 were designated as potential interactors of pUL56.

#### Recombinant protein expression and purification

For bacterial recombinant expression, the cytoplasmic region (residues 1-207) of UL56 from HSV-1 strain KOS was cloned into a vector derived from pOPT (Teo et al., 2004) encoding *Schistosoma japonicum* GST followed by a human rhinovirus 3C cleavage sequence fused to the N terminus and LysHis_6_ fused to the C terminus (GST-UL56(1-207)-His). Full-length (residues 1-454) and truncated forms (residues 1-362, 27-362, 27-275, 276-362 and 27-236) of GOPC (UniProt ID Q9HD26-2) were cloned from HeLa cell cDNA into a vector derived from pOPT (Teo et al., 2004) encoding a MetAlaHis_6_ tag fused to the N terminus of each construct (His-GOPC).

His-GOPC (both full-length and truncations) was expressed in *Escherichia coli* BL21(DE3)pLysS cells (Novagen) and GST-UL56(1–207)-His was expressed in *E. coli* T7 Express LysY/Iq cells (New England Biolabs). Cells were cultured in 2 × TY medium to an OD600 between 0.8 and 1.0. For His-GOPC, the culture was cooled to 22°C before adding 0.2 mM IPTG and culturing for a further 16 h. For GST-UL56(1–207)-His, 1 mM IPTG was added and the cells were cultured for a further 2 h. Cells were harvested by centrifugation and pellets stored at −80°C.

For His-GOPC, cell pellets were resuspended on ice in Ni^2+^ wash buffer (20 mM Tris pH 7.5, 20 mM Imidazole, 500mM NaCl) supplemented with 0.5 mM MgCl_2_, 1.4 mM 2-mercaptoethanol, 0.05% TWEEN-20, 400 U Bovine DNase I and 200 μL EDTA-free protease inhibitors (Sigma-Aldrich) and lysed by passing through a TS series cells disruptor (Constant Systems) at 24 kpsi. Lysates were cleared by centrifugation (40,000 × g, 30 min, 4°C) and incubated with NiNTA agarose (QIAGEN) pre-equilibrated in Ni^2+^ wash buffer for 60 min at 4°C. The resin was washed with > 20 column volumes (cv) of Ni^2+^ wash buffer and protein was eluted in Ni^2+^ elution buffer (20 mM Tris pH 7.5, 250 mM imidazole, 500mM NaCl) before being concentrated and applied to a Superdex 200 16/600 gel filtration column (GE Healthcare) that had been pre-equilibrated in gel filtration buffer (20 mM Tris, 200 mM NaCl, 1 mM DTT) at room temperature. Eluted fractions containing purified His-GOPC were pooled, concentrated and small (< 100 μL) aliquots were snap-frozen in liquid nitrogen for storage at −80°C.

For GST-UL56(1-207)-His, cells were resuspended on ice in 50 mM sodium phosphate pH 7.6, 300 mM NaCl, 0.5 mM MgCl_2_, 1.4 mM 2-mercaptoethanol, 0.05% TWEEN-20, 400 U Bovine DNase I and 200 μL EDTA-free protease inhibitors (Sigma-Aldrich) before lysis and clarification as described above. Cleared lysates were incubated with glutathione Sepharose 4B (GE Life Science) that had been pre-equilibrated in GSH wash buffer (50 mM sodium phosphate pH 7.6, 300 mM NaCl, 1 mM DTT) for 1 h at 4°C. The resin was washed with 10 cv of GSH wash buffer before being resuspended in 20 cv of 25 mM sodium phosphate pH 7.5, 150 mM NaCl, 1 MgCl_2_, 0.5 mM DTT and incubated at room temperature for 30 min with 50 U/mL benzonase nuclease (Sigma-Aldrich) to digest co-purifying nucleic acids. The resin was then washed with 20 cv of 50 mM sodium phosphate pH 7.6, 1 M NaCl to remove residual nucleotide binding before being washed with a further 40 cv of GSH wash buffer. Protein was eluted using GSH wash buffer supplemented with 25 mM reduced glutathione. The protein was then captured using NiNTA agarose that had been equilibrated in Ni^2+^ wash buffer, the resin was washed with > 20 cv of Ni^2+^ wash buffer, and the protein eluted in Ni^2+^ elution buffer before being injected onto a 10/300 Superdex 200 gel filtration column (GE Healthcare) equilibrated in gel filtration buffer (as above). Eluted fractions containing UL56 were pooled, concentrated and snap-frozen in small (< 100 μL) aliquots for storage at −80°C.

#### Protein GST pull-down assays

Bait proteins were diluted to 5 μM in pull-down buffer (20 mM Tris pH 7.5, 200 mM NaCl, 0.1% NP-40, 1 mM DTT, 1 mM EDTA) and, for each experiment, 200 μL of bait mixture was incubated for 15-30 min at room temperature with 10 μL of glutathione magnetic beads (Pierce) that had been pre-equilibrated in pull-down buffer. Supernatant was removed and resin was washed twice with pull-down buffer. Bait-loaded resin was incubated with purified His-GOPC (full-length or truncated) or clathrin N-terminal domain (Muenzner et al., 2017) diluted to 10 μM in pull-down buffer for 60 min at room temperature in a final volume of 200 μL per experiment. Unbound prey was removed and the beads washed four times with pull-down buffer. Bound proteins were eluted using pull-down buffer supplemented with 50 mM reduced glutathione. Samples were resolved by SDS-PAGE and visualized using InstantBlue Coomassie stain (Expedeon).

#### Immunofluorescence microscopy

Cells were seeded to be a third confluent on #1.5 glass coverslips and transfected with TransIT-LT1 or infected at MOI of 1 with crude virus stocks in complete media. Where indicated, cells were treated with 10 μM MG132 or an equivalent volume of carrier (DMSO). Plasmids expressing EGFP-tagged pUL56 or pUL56-AAXA were used for pUL56-GOPC co-localization analysis. Plasmids expressing FLAG-tagged TLR2 and pUL56 or pUL56-AAXA were used for TLR2 localization analysis. At 1 day post-transfection or 6 hpi, the samples were fixed by incubation with 3% (v/v) electron microscopy-grade formaldehyde (PFA, Polysciences) in PBS for 15 min at room temperature (Figures 4B, 6D, and S1A) or by incubation with ice-cold 250 mM HEPES pH 7.5, 4% (v/v) PFA for 5 min, incubation with 250 mM HEPES pH 7.5, 8% (v/v) PFA at room temperature for 10 min, washing with PBS and incubation with 25 mM NH_4_Cl for 5 min (Figure 4C). For surface TLR2 detection, cells were incubated with anti-FLAG antibody for 1 h at 37°C prior to fixation. Cells were permeabilized and washed using PBS supplemented with 1% (v/v) FBS, 0.1% Triton X-100 (Figures 4B, 6D, and S1A) or 0.1% saponin (Figure 4C). For staining of infected cells where the primary antibody was from a rabbit, a 2 h blocking step using PBS supplemented with 100 μg/mL human IgG (Sigma-Aldrich), 10% (v/v) FBS was included before incubation with the primary antibody. Antibodies were diluted into PBS plus 10% (v/v) FBS supplemented with 100 μg/mL human IgG for staining of infected cells using antibodies raised in rabbit (Figure 4B), or PBS plus 10% (v/v) FBS supplemented with 0.1% saponin (Figure 4C), or PBS plus 1% (v/v) FBS, supplemented with 0.1% Triton X-100 (Figures 6D and S1A). After immunostaining, the coverslips were mounted with ProLong Gold Antifade Mountant containing 4’,6-diamidino-2-phenylindole (DAPI) (ThermoFisher) (Figures 4B, 6D, and S1A) or with Mowiol 4-88 (Merck) containing 200 nM DAPI (Figure 4C). For Figures 4B, 6D, and S1A, samples were analyzed with an inverted Olympus IX81 widefield microscope. Illumination was performed with a Lumen 200 arc lamp (Prior Scientific) and bandpass filters for DAPI (excitation of 350/50 nm and emission of 455/50 nm), Alexa Fluor 488 (excitation of 490/20 nm and emission of 525/36 nm), and Alexa Fluor 568 (excitation of 572/35 nm and emission of 605/52 nm) (Chroma Technology Corp). Images were acquired with Image-Pro Plus software (Media Cybernetics), a Retiga EXi Fast1394 interline CCD camera (QImaging), and a 60 × Plan Apochromat N oil objective (numerical aperture 1.42) (Olympus) for a pixel resolution of 107.5 nm/pixel. For Figure 4C, images were acquired using a Zeiss LSM780 confocal laser scanning microscopy system mounted on an AxioObserver.Z1 inverted microscope using a 64 × Plan Apochromat oil objective (numerical aperture 1.4).

#### Virus growth curves, and plaque assays

Growth curves were performed using HaCaT cells infected in complete media with crude virus stocks of HSV-1 WT or HSV-1 ΔUL56 at MOI of 10. After adsorption for 1 h at 37°C, cells were incubated with acid wash (40 mM citric acid, 135 mM NaCl, 10 mM KCl; pH 3.0) for 1 min and washed 3x with PBS before cell culture media was added back. The time of acid wash was deemed 0 hpi. At various times post-infection, cells were harvested by freezing the plate at −70°C. After freezing the last time point, samples were freeze-thawed together 2 subsequent times and scraped before they were titered. Titrations were performed on Vero monolayers. Cells were inoculated with serial dilutions of the samples for 1 h, after which DMEM containing 0.3% high viscosity carboxymethyl cellulose, 0.3% low viscosity carboxymethyl cellulose, 2% (v/v) FBS, 2 mM L-glutamine, 100 U/mL penicillin, and 100 μg/mL streptomycin was overlaid. After 3 days, cells were fixed in 3.75% (v/v) formaldehyde in PBS for 30 min and stained with 0.1% toluidine blue.

For plaque size measurements, HaCaT, HFF hTERT, or Vero cells were grown in 6-well plates. The cells were infected and fixed as described above, but they were stained with an anti-gD antibody (LP2). Plaques were visualized with a secondary antibody conjugated to horseradish peroxidase and the DAB peroxidase substrate following the manufacturer’s instructions (Vector Laboratories). Plaques were scanned at 300 dpi and plaque diameters were measured with ImageJ (https://imagej.nih.gov/ij/).

#### Generation of CRISPR knockout HaCaT cells

HaCaT cells were seeded at 50% confluence and transfected with the PX459 CRISPR plasmid containing relevant guide RNAs (GOPC 1: GGAACATGGATACCCCGCCA; GOPC 2: GAGAGATCGATCCAGACCAAG) and Lipofectamine 2000 according to the manufacturer’s instructions. pSpCas9(BB)-2A-Puro (PX459) V2.0 was a gift from Feng Zhang (Addgene plasmid # 62988; http://n2t.net/addgene:62988; RRID:Addgene_62988) (Ran et al., 2013). One day post-transfection the medium was changed to contain 2 μg/mL puromycin, and 3 days post-transfection the medium was changed to selection-free medium. Clonal cell lines were expanded and tested for loss of GOPC by western blot analysis and genomic sequencing.

#### Flow Cytometry

HaCaT cells infected with crude virus stocks were washed 2 times with PBS and detached with accutase (Sigma-Aldrich). Cells were pelleted at 400 × g for 5 min and washed once with PBS containing 2% (v/v) FBS. For extracellular staining, cells were stained with anti-human CD282 (TLR2) antibody (BioLegend, 153003) and incubated for 1 h at room temperature. Stained cells were washed once and fixed in 4% (v/v) formaldehyde in PBS for 20 min at room temperature. Data was acquired with a FACSCalibur and analyzed with Flowing Software version 2.5.1 (http://flowingsoftware.btk.fi/).

### Quantification and Statistical Analysis

For TMT-based proteomic data statistical analysis Significance B was used to estimate the probability that each ratio was significantly different to 1 (Cox and Mann, 2008). Values were calculated and corrected for multiple hypothesis testing using the method of Benjamini-Hochberg in Perseus version 1.5.1.6 (Cox and Mann, 2008). A corrected p value < 0.05 was considered statistically significant. Hierarchical centroid clustering based on uncentered Pearson correlation of data normalized by comparing the signal:noise values to the average mock-infection were performed using Cluster 3.0 (Stanford University) (de Hoon et al., 2004), and visualized using Java Treeview (http://jtreeview.sourceforge.net) (Saldanha, 2004). For analysis of temporal classes, viral protein expression was normalized and subjected to K-means analysis using XLSTAT base (Addinsoft, version 18.06) and clustered with 1-15 classes.

For SILAC-based plasma membrane data statistical analysis hierarchical centroid clustering based on uncentered Pearson correlation of the normalized ratios generated by MaxQuant was performed using Cluster 3.0 (Stanford University) (de Hoon et al., 2004) and visualized with Java Treeview (http://jtreeview.sourceforge.net) (Saldanha, 2004).

For SILAC-IP data statistical analysis a one-sample, two-sided t test with a threshold p value of 0.05 was performed on each group to identify significantly enriched proteins. Proteins with a log_2_ fold change greater than 1 and a p value smaller than 0.05 were designated as potential interactors.

## Supplemental Information

Document S1. Figures S1–S6

Table S1. Quantitative Temporal Analysis of HSV Infection, Related to Figure 1 Interactive spreadsheet of the quantitative temporal viromics data from the HSV-1 whole cell lysate (WCL) time course. The “Data” worksheet shows minimally annotated protein data, with only formatting and normalization modifying the raw data. The “Plotter” worksheet enables generation of individual protein abundance changes from the time course. The “Viral classes” worksheet shows the K-means clusters of all canonical HSV-1 proteins, a list of 5 clusters is shown. The “MS Quantification” worksheet shows the number of proteins and peptides quantified. The TMT reagents used to label samples are detailed in the “TMT labelling” worksheet. The “Novel 6FT-ORFs” worksheet contains details of putative additional HSV-1 proteins that increased in abundance over the course of infection.

Table S2. Comparative Analysis of Whole-Cell Protein, Total RNA, Newly Synthesized RNA, and Ribosome Profiling Data from HSV-1-Infected Cells, Related to Figure 2 Data from the HSV-1 whole cell lysate time course (Figure 1) was compared to RNA sequencing (total RNA and newly synthesized RNA) and ribosome profiling data from a recent study (Rutkowski et al., 2015). The “Plotter” worksheet includes interactive graphs displaying fold change at the latest infection time point for each dataset. The “WCL data” worksheet shows normalized values for all proteins quantified. The “Normalized RNA and RP” includes RNA sequencing and ribosome profiling data that has been normalized in an identical fashion to that described for the proteomics data. The “WCL 18 h vs RNA 8 h” worksheet includes all data from each study and compares fold changes at the latest time points for each dataset.

Table S3. Manipulation of Host-Cell Pathways during HSV Infection, Related to Figure 2 DAVID functional enrichment analysis for proteins downregulated > 2-fold compared to a background of all proteins quantified. Only significantly enriched clusters are shown with Benjamini-Hochberg corrected p < 0.05. There were no significant clusters among proteins upregulated > 2-fold.

Table S4. Identification of Cellular Interaction Partners of pUL56, Related to Figure 3 Spreadsheet listing the SILAC ratios and statistical analysis of proteins quantified in pull-downs of pUL56 followed by mass spectrometry (IP-MS). Two different constructs of pUL56 encompassing either the full-length protein or its cytoplasmic domain were tested and the respective results are listed in separate tabs.

Table S5. Identification of pUL56 Degradation Targets, Related to Figure 5 Interactive spreadsheet displaying whole cell protein changes between cells infected with HSV-1 WT, HSV-1 ΔUL56 or mock. The “Data” worksheet shows minimally annotated protein data, with only formatting and normalization modifying the raw data. The “Plotter” worksheet enables generation of individual protein abundance changes, comparing the different viruses and time points. The “MS Quantification” worksheet shows the number of proteins and peptides quantified. The TMT reagents used to label samples are detailed in the “TMT labelling” worksheet. The “Novel 6FT-ORFs” worksheet contains details of putative additional HSV-1 proteins that increased in abundance over the course of infection.

Table S6. Analysis of the Plasma Membrane Proteome in Infected Cells, Related to Figure 6 Interactive spreadsheet of plasma membrane protein changes between cells infected with HSV-1 WT, HSV-1 ΔUL56 or mock. The “Data” worksheet shows minimally annotated protein data, with formatting and normalization modifying the raw data. Gene Ontology terms were used to identify proteins associated with the plasma membrane, as described in the text. The “Plotter” worksheet enables generation of individual protein ratios between the three conditions. The ‘MS Quantification’ worksheet shows the number of proteins and peptides quantified.

Table S7. Comparative Analysis of Whole-Cell Protein and Plasma Membrane Protein Changes Caused by HSV-1 Infection, Related to Figures 1 and 6 Comparative analysis of data from whole cell proteomics and plasma membrane proteomics at 6 hpi. The “Plotter” worksheet generates graphs comparing protein fold changes for each experiment. Proteins quantified in either experiment are shown in the “WCL and PMP combined” worksheet. Whole cell protein and plasma membrane protein changes can be compared in the “Quantified in both” worksheet. Proteins downregulated > 1.5-fold by HSV-1 infection are included in either of the two “Downregulated” worksheets, depending on whether they were downregulated in both whole cell and plasma membrane experiments or in plasma membrane proteomics alone.

Table S8. Analysis of the Plasma Membrane Proteome in GOPC-Knockout Cells, Related to Figure 7 The “Plotter” worksheet generates graphs comparing relative protein abundance in wild type (WT) and GOPC-knockout HaCaT cells. The “Data” worksheet shows normalized data for all proteins quantified and includes Gene Ontology annotations. The TMT reagents used to label samples are detailed in the “TMT labelling” worksheet. The overall number of proteins and peptides quantified in the experiment are included in the “MS Quantification” worksheet.

Document S2. Article plus Supplemental Information
